# The Efficacy and Safety of (Neo)Adjuvant Therapy for Gastric Cancer: A Network Meta-analysis

**DOI:** 10.3390/cancers11010080

**Published:** 2019-01-11

**Authors:** Tom van den Ende, Emil ter Veer, Mélanie Machiels, Rosa M. A. Mali, Frank A. Abe Nijenhuis, Laura de Waal, Marety Laarman, Suzanne S. Gisbertz, Maarten C. C. M. Hulshof, Martijn G. H. van Oijen, Hanneke W. M. van Laarhoven

**Affiliations:** 1 Department of Medical Oncology, Cancer Center Amsterdam, Amsterdam University Medical Centers (UMC) location AMC, University of Amsterdam, 1105 AZ Amsterdam, The Netherlands; t.vandenende@amc.uva.nl (T.v.d.E.); e.terveer@amc.uva.nl (E.t.V.); rosa.mali@hotmail.com (R.M.A.M.); f.a.abenijenhuis@amc.uva.nl (F.A.A.N.); l.dewaal@amc.uva.nl (L.d.W.); m.laarman@amc.uva.nl (M.L.); m.g.vanoijen@amc.uva.nl (M.G.H.v.O.); 2 Department of Radiotherapy, Cancer Center Amsterdam, Amsterdam University Medical Centers (UMC) location AMC, University of Amsterdam, 1105 AZ Amsterdam, The Netherlands; m.machiels@amc.uva.nl (M.M.); m.c.hulshof@amc.uva.nl (M.C.C.M.H.); 3 Department of Surgery, Cancer Center Amsterdam, Amsterdam University Medical Centers (UMC) location AMC, University of Amsterdam, 1105 AZ Amsterdam, The Netherlands; s.s.gisbertz@amc.uva.nl

**Keywords:** stomach neoplasms, chemotherapy, chemoradiotherapy, perioperative

## Abstract

*Background*: Alternatives in treatment-strategies exist for resectable gastric cancer. Our aims were: (1) to assess the benefit of perioperative, neoadjuvant and adjuvant treatment-strategies and (2) to determine the optimal adjuvant regimen for gastric cancer treated with curative intent. *Methods*: PubMed, EMBASE, CENTRAL, and ASCO/ESMO conferences were searched up to August 2017 for randomized-controlled-trials on the curative treatment of resectable gastric cancer. We performed two network-meta-analyses (NMA). NMA-1 compared perioperative, neoadjuvant and adjuvant strategies only if there was a direct comparison. NMA-2 compared different adjuvant chemo(radio)therapy regimens, after curative resection. Overall-survival (OS) and disease-free-survival (DFS) were analyzed using random-effects NMA on the hazard ratio (HR)-scale and calculated as combined HRs and 95% credible intervals (95% CrIs). *Results*: NMA-1 consisted of 9 direct comparisons between strategies for OS (14 studies, *n* = 4187 patients). NMA-2 consisted of 16 direct comparisons between adjuvant chemotherapy/chemoradiotherapy regimens for OS (37 studies, *n* = 10,761) and 14 for DFS (30 studies, *n* = 9714 patients). Compared to taxane-based-perioperative-chemotherapy, surgery-alone (HR = 0.58, 95% CrI = 0.38–0.91) and perioperative-chemotherapy regimens without a taxane (HR = 0.79, 95% CrI = 0.58–1.15) were inferior in OS. After curative-resection, the doublet oxaliplatin-fluoropyrimidine (for one-year) was the most efficacious adjuvant regimen in OS (HR = 0.47, 95% CrI = 0.28–0.80). *Conclusions*: For resectable gastric cancer, (1) taxane-based perioperative-chemotherapy was the most promising treatment strategy; and (2) adjuvant oxaliplatin-fluoropyrimidine was the most promising regimen after curative resection. More research is warranted to confirm or reproach these findings.

## 1. Introduction

Gastric adenocarcinoma is one of the leading causes of cancer related mortality on a global scale [1]. Even after a curative resection, relapse-related death remains a major problem. There is no global consensus on the optimum treatment strategy (perioperative, neoadjuvant or adjuvant systemic therapy and/or radiotherapy) to be administered in addition to surgery for resectable gastric cancer. Perioperative chemotherapy is the preferred treatment strategy in many countries in Europe, as there is evidence this will reduce the number of relapses [2]. For a decade, the perioperative anthracycline-based MAGIC regimen, consisting of epirubicin, cisplatin and 5-FU was the preferred option [3]. Recently, the FLOT-4 trial established the superiority of a perioperative taxane-based regimen with docetaxel, oxaliplatin and 5-FU with leucovorin (FLOT) over perioperative epirubicin, cisplatin and a fluoropyrimidine; 5-FU or capecitabine (ECF/ECX) [4]. The FLOT regimen significantly improved survival (median 50 months with FLOT and 35 months with ECF/ECX) and led to a higher number of R0 resections (84% with FLOT and 77% with MAGIC) [4]. In Asian countries, after a curative resection, adjuvant chemotherapy, usually without any neoadjuvant therapy, is the standard of care [5]. For example, adjuvant oxaliplatin combined with capecitabine or S-1 as monotherapy after curative resection are two established treatment regimens [5]. Finally, in the United States adjuvant chemotherapy with radiotherapy after curative resection is a frequently applied treatment strategy, based on the intergroup 0116 trial [6]. However, the American NCCN guideline also acknowledges the benefit of the other treatment strategies, including perioperative and adjuvant chemotherapy [7].

After the landmark MAGIC trial, neoadjuvant and perioperative strategies were more frequently applied to improve overall survival [3]. In perioperative trials, only half of all patients start with adjuvant therapy after a surgical resection [3,4]. The question rises whether administration of neoadjuvant, or adjuvant therapy only would lead to the same survival benefit as a perioperative regimen. Moreover, the optimal adjuvant regimen after a curative resection has not yet been established. Network meta-analysis (NMA) allows for the comparison of more than two treatments at once by introducing a common comparator (e.g., surgery only) and combining direct with indirect estimates into a combined effect size [8,9]. Therefore, NMA can aid clinical decision making by comparing different regimens with one or multiple comparators, even if studies comparing regimens head-to-head are not available. NMA can also aid in finding the optimum treatment backbone for future randomized trials.

We conducted a systematic review, using NMA, with two primary aims regarding efficacy: (1) compare the clinical benefit of perioperative, neoadjuvant and adjuvant treatment strategies; and (2) to establish the optimal adjuvant regimen after a curative resection for gastric cancer. Our secondary aim was to investigate the safety of different chemo(radio)therapy regimens.

## 2. Results

### 2.1. Description of the Included Studies

**Figure 1:**
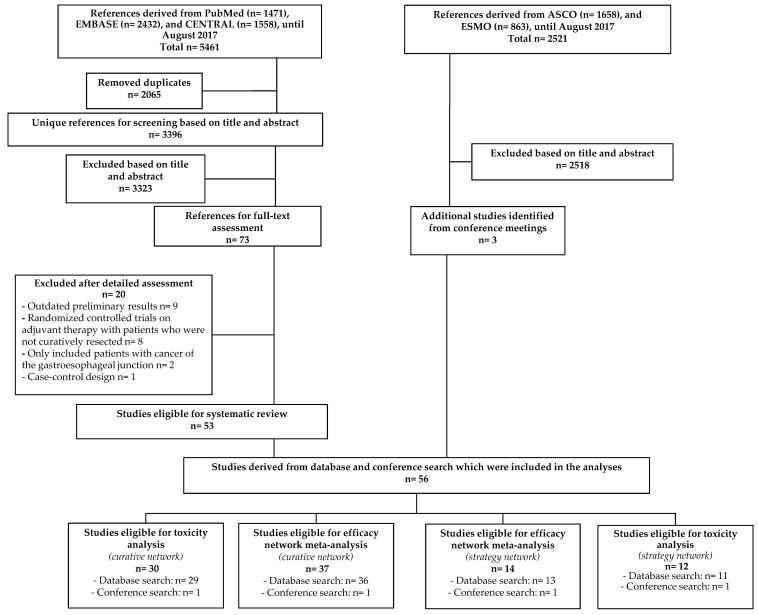
Flowchart of references derived from database search (left) and from conference search (right). Due to the absence of enough data to calculate a hazard ratio for survival, three studies on different treatment strategies and two studies on adjuvant therapy after curative resection were only eligible for the toxicity analyses. *N* = number of studies.

From a total of 5461 unique references, identified by searching PubMed, Embase and Central, 73 references remained after title and abstract screening. 20 references were excluded after full text assessment including the SAMIT trial for the primary analysis as it included R1 resected patients [10]. The results of the SAMIT trial were only used for a sensitivity analysis. By searching the conference meetings of the ASCO and ESMO meetings three additional studies were identified. In total, 56 studies (*n* = 15,795 patients) could be included in any of the analyses (Figure 1). Two separate networks were created, one comparing different treatment strategies; perioperative, neoadjuvant and adjuvant therapy and one in which different adjuvant regimens were compared after a curative resection. Before merging different treatment strategies or drug classes, a preliminary NMA was conducted for both networks. When taxane-based neoadjuvant and taxane-based adjuvant chemotherapy were separated from non-taxane containing neoadjuvant/adjuvant chemotherapy the network lost the ability to detect any significant difference between comparisons (Figures S1 and S2). Therefore, due to the low amount of studies and patients for each comparison neoadjuvant regimens were pooled together as well as different adjuvant regimens. Based on the FLOT-4 trial, taxane-based perioperative chemotherapy was kept as a separate clinical entity compared to non-taxane containing perioperative chemotherapy [4]. A description of the baseline characteristics (Table 1 and Table 2), pairwise meta-analyses (Figures S3–S5) and risk of bias of both NMAs can be found in the Supplementary Results (Figures S6 and S7).

**Figure 2:**
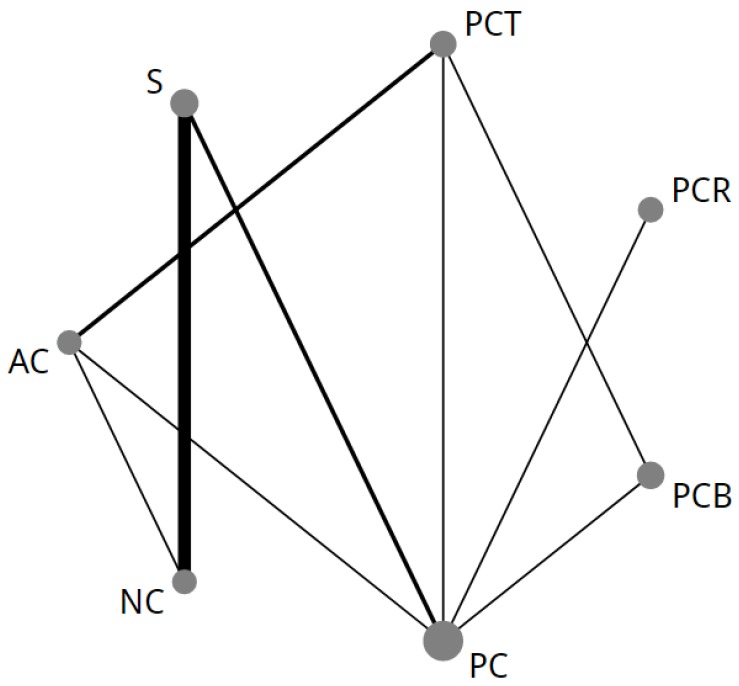
First network of all treatments in the strategy network meta-analysis (NMA-1). The size of each node corresponds to the number of patients who were randomly assigned to receive the given regimen. The lines connect the regimens that were directly compared in head-to-head randomized controlled trials (RCTs). The thickness of the lines corresponds to the number of RCTs. AC = adjuvant chemotherapy; NC = neoadjuvant chemotherapy; PC = perioperative chemotherapy without a taxane; PCB = perioperative chemotherapy combined with bevacizumab; PCR = perioperative chemotherapy combined with adjuvant chemoradiotherapy; PCT = taxane-based perioperative chemotherapy; S = surgery only.

The NMA comparing treatment strategies (NMA-1) consisted of 14 individual studies [3,4,11,12,13,14,15,16,17,18,19,20,21,22] and seven different treatment strategies (Figure 2). For OS there were nine direct comparisons (*n* = 4187 patients). For one study, the HR for OS was extracted from a previously conducted meta-analysis [17,60]. There was insufficient data available to conduct a NMA for progression free survival or disease free survival.

**Figure 3:**
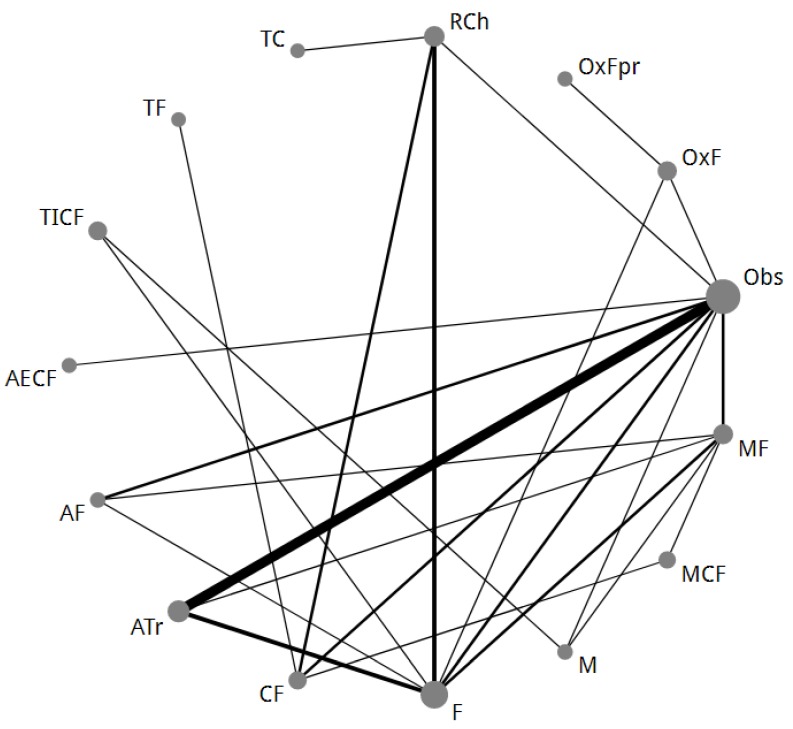
Second network of all different treatment regimens in the adjuvant therapy for curatively resected gastric cancer network meta-analysis (NMA-2). The size of each node corresponds to the number of patients who were randomly assigned to receive the given regimen. The lines connect the regimens that were directly compared in head-to-head randomized controlled trials (RCTs). The thickness of the lines corresponds to the number of RCTs. A = anthracycline; ATr = anthracycline-based triplet; C = cisplatin; E = etoposide; F = fluoropyrimidine; I = irinotecan; M = mitomycin C; Obs = observation; Ox = oxaliplatin; OxFpr = eight cycles of oxaliplatin-fluoropyrimidine thereafter eight cycles of fluoropyrimidine monotherapy; RCh = chemoradiotherapy; T = taxane.

After merging, a total of 37 studies [6,24,25,26,27,28,29,30,31,32,33,34,35,36,37,38,39,40,41,42,43,44,45,46,47,48,49,50,51,52,53,54,55,56,57,58,59] were included in the NMA comparing adjuvant therapy after curative resection (NMA-2), with 14 different radio/chemotherapy regimens. For OS, there were 16 direct comparisons between different regimens with in total *n* = 10,761 patients (Figure 3). For DFS, there were 14 direct comparisons with in total *n* = 9714 patients. There was not enough data available to calculate the HR for OS and DFS in the published reports of seven RCTs [25,30,31,32,42,51,55] and therefore, HRs were extracted from a previously conducted individual patient data meta-analysis or a meta-analysis [61,62].

### 2.2. NMA-1 Comparing Different Treatment Strategies 

**Figure 4:**
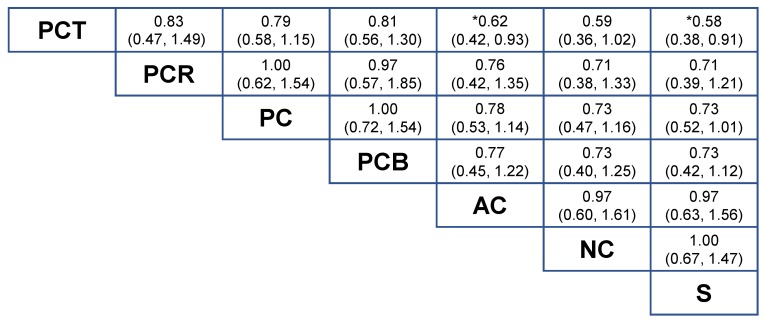
Results of the treatment-strategy random effects network meta-analysis (NMA-1) for seven different strategies in terms of overall survival. Relative effects in combined hazard ratios and 95% credible intervals are shown for the combination chemotherapy regimens. The hazard ratio for a given comparison could be read in the intersection of two treatments. The strategies are grouped according to their baseline efficacy compared with surgery-alone. All z-tests to compare two treatments were performed two-sided. * *p* < 0.05. Abbreviations: AC = adjuvant chemotherapy; NC = neoadjuvant chemotherapy; PC = perioperative chemotherapy regimens without a taxane; PCB = perioperative chemotherapy combined with bevacizumab; PCR = perioperative chemotherapy combined with adjuvant chemoradiotherapy; PCT = taxane-based perioperative chemotherapy; S = surgery only.

OS could be compared in the strategy-based NMA-1 (Figure 4). Taxane-based perioperative chemotherapy (PCT) was the most effective treatment strategy compared to surgery alone (S), HR = 0.58 (95% CrI = 0.38 to 0.91). Taxane-based perioperative chemotherapy was superior compared to adjuvant chemotherapy (AC), HR = 0.62 (95% CrI = 0.42 to 0.93) and was non-significant compared to neoadjuvant chemotherapy (NC), HR = 0.59 (95% CrI = 0.36 to 1.02) although a clinically-relevant HR was found (HR < 0.80). Compared to perioperative chemotherapy without a taxane (PC), the addition of adjuvant chemoradiotherapy (PCR), HR = 1.00 (95% CrI = 0.62 to 1.54) or bevacizumab to perioperative chemotherapy (PCB), HR = 1.00 (95% CrI = 0.72 to 1.54) did not result in a survival benefit. Compared to surgery-alone, no survival benefit was found for neoadjuvant chemotherapy, HR = 1.00 (95% CrI = 0.67 to 1.47) nor for adjuvant chemotherapy, HR = 0.97 (95% CrI = 0.63 to 1.56).

### 2.3. NMA-2 Comparing Adjuvant Regimens after Curative Resection

**Figure 5:**
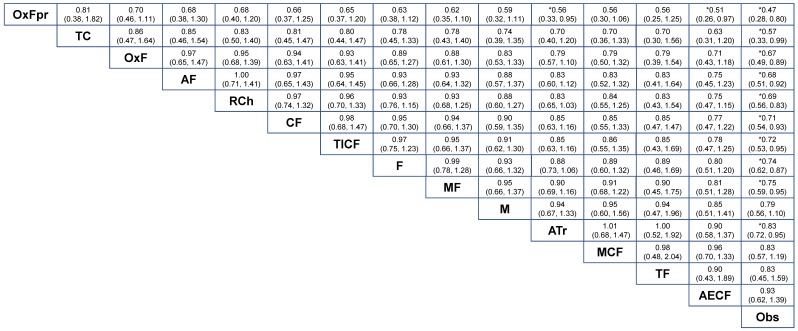
Results of the adjuvant therapy for curatively resected gastric cancer random effects network meta-analysis (NMA-2) for 14 different treatment modalities in terms of overall survival. Relative effects in combined hazard ratios and 95% credible intervals are shown for the combination chemotherapy regimens. The hazard ratio for a given comparison could be read in the intersection of two treatments. The strategies are grouped according to their baseline efficacy compared with observation-alone. All z-tests to compare two treatments were performed two-sided. * *p* < 0.05. Abbreviations: A = anthracycline; ATr = anthracycline-based triplet; C = cisplatin; E = etoposide; F = fluoropyrimidine; I = irinotecan; M = mitomycin C; Obs = observation; Ox = oxaliplatin; OxFpr = eight cycles of oxaliplatin-fluoropyrimidine thereafter eight cycles of fluoropyrimidine monotherapy; RCh = chemoradiotherapy; T = taxane.

**Figure 6:**
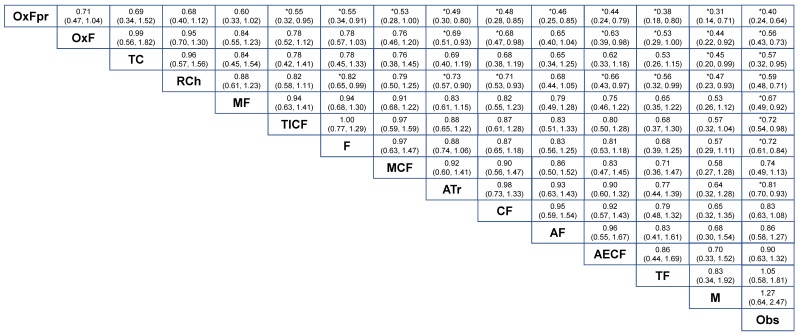
Results of the adjuvant therapy for curatively resected gastric cancer random effects network meta-analysis (NMA-2) for 14 different treatment modalities in terms of disease free survival. Relative effects in combined hazard ratios and 95% credible intervals are shown for the combination chemotherapy regimens. The hazard ratio for a given comparison could be read in the intersection of two treatments. The strategies are grouped according to their baseline efficacy compared with observation-alone. All z-tests to compare two treatments were performed two-sided. * *p* < 0.05. Abbreviations: A = anthracycline; ATr = anthracycline-based triplet; C = cisplatin; E = etoposide; F = fluoropyrimidine; I = irinotecan; M = mitomycin C; Obs = observation; Ox = oxaliplatin; OxFpr = eight cycles of oxaliplatin-fluoropyrimidine thereafter eight cycles of fluoropyrimidine monotherapy; RCh = chemoradiotherapy; T = Taxane.

The results for OS and DFS for NMA-2 are summarized in Figure 5 and Figure 6. Compared with observation-alone (Obs), the largest survival benefit was found for oxaliplatin with a prolonged 1-year course of a fluoropyrimidine (OxF-prolonged) which reached a HR = 0.47 (95% CrI = 0.28 to 0.80) for OS and HR = 0.40 (95% CrI = 0.24 to 0.64) for DFS. OxF-prolonged showed a non-significant but clinically-relevant HR to fluoropyrimidine-monotherapy (F), HR = 0.63 (95% CrI = 0.38 to 1.12) in OS. In addition, OxF-prolonged was more effective in terms of DFS compared to fluoropyrimidine monotherapy, HR = 0.55 (95% CrI = 0.34 to 0.91). OxF showed superior efficacy compared to a cisplatin-fluoropyrimidine doublet (CF) in DFS, HR = 0.68 (95% CrI = 0.47 to 0.98) but not in OS (Figure 5). Increased efficacy was found for OxF-prolonged compared to an anthracycline-based triplet (ATr) in terms of both OS, HR = 0.56 (95% CrI = 0.33 to 0.95) and DFS, HR = 0.49 (95% CrI = 0.30 to 0.80). Radiotherapy combined with chemotherapy (RCh) showed no benefit compared to OxF-prolonged, OxF or a taxane-cisplatin doublet (TC) in the OS and DFS analysis (Figure 5 and Figure 6).

### 2.4. Network Consistency and Sensitivity Analyses 

An extended description of the assessment of network inconsistency and the comparison between direct and combined HRs can be found in the Supplementary Results (Figures S8–S10). Node-split models were non-significant for both NMAs. For NMA-1, perioperative trials were mainly studied in a Western population. Sensitivity analysis for descent, stage and type of lymph node dissection showed the same overall trend indicating perioperative chemotherapy with a taxane is the most promising treatment strategy. However, it must be taken into account the sensitivity analyses for NMA-1 were relatively underpowered due to the low amount of studies per sensitivity analysis. For NMA-2 oxaliplatin containing regimens were only studied in Asian D2 dissected patients. For the other regimens, sensitivity analyses for descent, stage and type of lymph node dissection did not have a major impact on the direction of the HR. For NMA-2, when the results of the comparison between fluoropyrimidine monotherapy and sequential therapy with a fluoropyrimidine and a taxane (TF) were added from the SAMIT trial, which included 7% R1 resected patients, TF reached a significant HR = 0.71 (95% CrI = 0.54 to 0.93) for OS compared to observation. 

### 2.5. Toxicity and Surgical Complications

In total, 12 studies for the treatment strategy NMA-1 contributed to the grade 3–4 toxicity and surgical related adverse events (AEs) pair-wise meta-analyses. For the NMA-2 comparing adjuvant therapy after curative resection 30 studies were included in the grade 3–4 toxicity AEs pair-wise meta-analyses. For NMA-2 only regimens which were significant (*p* < 0.05) compared to observation-alone were included in the grade 3–4 AE analyses. Preoperative TOxF showed an increased rate of neutropenia compared to preoperative ACF (52.3% and 40.0%, respectively, relative risk [RR] = 1.38, 95% CI = 1.05 to 1.81). However, preoperative ACF was associated with an increased rate of nausea and vomiting (26.3% and 12.5%, respectively, RR = 2.10, 95% CI = 1.23 to 3.60). Patients receiving bevacizumab in combination with perioperative ACF had an increased amount of anastomotic leakages compared to perioperative ACF (15.8% and 6.6%, respectively, RR = 2.40, 95% CI = 1.60 to 3.61). No significant increase in 30-day mortality or surgery related morbidity was found in patients which had received chemotherapy before the operation compared to patients which had received no treatment before surgery.

The pair-wise meta-analyses for adjuvant therapy after curative resection included six comparisons between chemotherapy/chemoradiotherapy and observation alone. Therefore, no RR could be calculated for these comparisons. The doublet oxaliplatin-fluoropyrimidine showed a more tolerable toxicity profile (OxF: neutropenia 22%; thrombocytopenia 8%; nausea/vomiting 15%; stomatitis 1%) than a cisplatin-fluoropyrimidine doublet (CF: neutropenia 26%; thrombocytopenia 14%; nausea/vomiting 30%; stomatitis 17%). S-1 monotherapy for one year had the lowest amount of grade 3–4 AEs (S-1: leukopenia 1%; anemia 1%; diarrhea 3%; stomatitis 0%). The addition of radiotherapy to a chemotherapeutic regimen did not significantly increase the amount of grade 3–4 AEs compared to the same chemotherapeutic backbone without radiotherapy. A full overview of grade 3–4 adverse events and surgical related outcomes can be found in the Supplementary Results (Tables S1–S3).

## 3. Discussion

Based on the results of our two NMAs for the comparison of treatment strategies and the comparison of adjuvant therapy after curative resection for resectable gastric cancer, three major conclusions can be drawn which may help guide clinical practice and future research. The results are mainly hypothesis-generating and should be interpreted accordingly as there are limitations associated with the performed analyses.

First, taxane-containing perioperative chemotherapy (PCT) was the most effective treatment strategy compared to surgery alone. Therefore, PCT is the preferred treatment strategy when patients have not yet received surgery and are sufficiently fit to start with chemotherapy. A meta-analysis, based in part on individual patient data, of 14 RCTs investigating the benefit of pre/perioperative chemo(radio)therapy for patients with gastroesophageal adenocarcinoma performed by the Cochrane group found a HR = 0.81 (95%CI 0.73–0.89, *p* < 0.0001) in favor of pre/perioperative therapy compared to surgery alone [63]. The different RCTs used relatively similar regimens based on platinum agents with or without anthracyclines. The Cochrane meta-analysis calculated a combined effect size for perioperative and neoadjuvant trials [63]. In NMA-1 we could separate perioperative from neoadjuvant trials and compared PCT with neoadjuvant therapy. The HR was in favor of PCT but did not reach statistical significance, HR = 0.59 (95% CrI 0.36–1.02). By using the NMA technique we could also compare PCT with adjuvant chemotherapy and found PCT to reach a statically significant survival benefit compared to adjuvant chemotherapy, HR = 0.62 (95% CrI 0.42–0.93). Perioperative chemotherapy without a taxane (PC) did not reach statistical significance in NMA-1 in the random effects model compared to surgery alone, HR = 0.73 (95% CrI 0.52–1.01). Although, it did reach statistical significance in the pairwise comparison between PC and surgery alone, HR = 0.73 (95% CrI 0.61–0.88), Figure S3. Our results do confirm the findings of the Cochrane review in favor of perioperative chemotherapy and we further identified the relative benefit of PCT compared to neoadjuvant or adjuvant chemotherapy. Moreover, perioperative chemotherapy is also an established treatment strategy in the ESMO and NCCN guidelines when patients have not yet received surgery [2,7]. Of note, approximately only 50% of the patients, in perioperative trials will start with adjuvant therapy [3,4]. Potentially, the administration of neoadjuvant chemotherapy could be as effective as perioperative chemotherapy. However, the findings of our NMA-1 suggest survival benefit may not solely be based on the administration of neoadjuvant therapy alone, as neoadjuvant chemotherapy failed to improve survival compared to surgery alone, HR = 1.00 (95% CrI = 0.67 to 1.47). Hypothetically, survival benefit can also be obtained by administering adjuvant chemotherapy after neoadjuvant therapy. Thus, rather than the timing of the chemotherapy, the amount of chemotherapy may be most relevant. Unfortunately, currently available data are insufficient to test this hypothesis and the results of our NMA should be interpreted with caution. The neoadjuvant and adjuvant arms in our strategy NMA-1 were relatively small and might thus be underpowered to detect a survival benefit for these strategies. For now, taxane-containing perioperative chemotherapy is preferable compared to neoadjuvant chemotherapy and adjuvant chemotherapy. A well powered randomized controlled trial should investigate if taxane-containing perioperative chemotherapy is superior compared to taxane-containing neoadjuvant chemotherapy.

Second, after a curative resection, the doublet oxaliplatin-fluoropyrimidine showed the largest survival benefit compared to observation-alone. The ESMO, NCCN and Japanese gastric cancer guidelines highlight the efficacy of a doublet containing oxaliplatin and fluoropyrimidine, based on the CLASSIC trial [2,5,64]. In Japan the use of S-1 as adjuvant therapy is considered to be a viable alternative, based on the ACTS-GS trial [65]. Results from NMA-2 indicated that oxaliplatin was preferable compared to cisplatin. Findings in advanced esophagogastric cancer support the use of oxaliplatin over cisplatin [66,67,68,69,70,71,72,73,74]. Also, the addition of oxaliplatin to a fluoropyrimidine in a prolonged-adjuvant treatment course conveyed survival benefit compared to fluoropyrimidine-monotherapy although OS results were non-significant due to a lack of power. Based on our NMA-2, the doublet oxaliplatin-fluoropyrmidine is preferred for patients in good condition and fluoro-pyrimidine-monotherapy should be reserved for patients with co-morbidity limiting intensive treatment.

Based on NMA-2, the use of anthracycline based chemotherapy is inferior to an oxaliplatin based doublet. This reflects results in advanced esophagogastric cancer where fluoropyrimidine doublets are preferred over cisplatin doublets and anthracycline-based triplets, as first-line treatment option [66,67]. Moreover, also for patients with esophageal cancer whom received neoadjuvant chemotherapy, anthracyclines in a triplet combination with cisplatin-fluoropyrimidine did not improve survival compared to the doublet cisplatin-fluoropyrimidine [75]. In sum, the addition of an anthracycline to a doublet regimen based on a platinum-fluoropyrimidine compound does not lead to additional survival benefit in esophagogastric cancer [76].

Third, currently there is no definitive advantage of incorporating adjuvant chemoradiotherapy in the curative treatment of gastric cancer. In NMA-1 adjuvant chemoradiotherapy combined with perioperative chemotherapy showed similar or even inferior efficacy compared to perioperative-taxane containing chemotherapy. Moreover, after a curative resection chemotherapy combined with radiotherapy did not improve survival compared to oxaliplatin-fluoropyrimidine or a taxane-cisplatin based doublet. The CRITICS study compared perioperative chemotherapy to perioperative chemotherapy combined with post-operative chemoradiotherapy and showed no benefit of post-operative chemoradiotherapy in any of the analyzed subgroups [13,77]. According to NMA-2, after a curative resection, the addition of radiotherapy to a chemotherapeutic regimen does not increase efficacy compared to chemotherapy-alone. However, chemoradiotherapy may be beneficial for patients with an R1 resection, according to data from the National Cancer Database [78]. Also, radiotherapy could be beneficial if an inadequate lymph node dissection (D0 or D1) was performed. In the intergroup 0116 trial, in which only 10% of the patients received a D2-dissection, benefit was observed from chemoradiotherapy compared to observation [6]. After a curative resection with an adequate D2 lymph node dissection, the ARTIST trial did not observe benefit from chemoradiotherapy compared to chemotherapy alone [54]. However, in a sub-analysis in node positive patients DFS was significantly better with chemoradiotherapy [54]. The ARTIST II trial (Clinical-Trials.gov identifier: NCT01761461) with node positive stage II and III gastric cancer patients (with an R0 and D2 lymph node dissection) is a three arm study comparing S-1 vs. Oxaliplatin + S-1 vs. Oxaliplatin+S-1 with radiotherapy and will confirm or reproach the results of our NMA.

Importantly, the CROSS study showed that neoadjuvant chemoradiotherapy in esophageal and GEJ cancer resulted in a significant survival benefit compared to surgery-alone [79]. To date, it is unknown if neoadjuvant chemoradiotherapy could improve outcomes in gastric cancer and results from ongoing randomized trials are eagerly awaited. The TOPGEAR (Clinical-Trials.gov identifier: NCT01924819), ESOPEC (Clinical-Trials.gov identifier: NCT02509286), Neo-AGIS (Clinical-Trials.gov identifier: NCT01726452) and the CRITICS II study (Clinical-Trials.gov identifier: NCT02931890) will all shed more light on the role of neoadjuvant chemoradiotherapy.

Our approach has some limitations. First, oxaliplatin-containing regimens were primarily studied in Asian, D2 lymph node dissected patients. Therefore, the results may only be extrapolated to the Western setting with caution. On the other hand, in Western countries oxaliplatin with a fluoropyrimidine is an established regimen for advanced esophagogastric cancer and curatively resected colon cancer [71,80].

Second, predictive factors could have influenced our results. In the perioperative chemotherapy trials gastric cancer, GEJ and esophageal adenocarcinoma patients were included which could have obscured the degree to which the results could be extrapolated to gastric cancer. Although, in the perioperative trials there was no significant heterogeneity in treatment effect according to tumor location [3,11]. Sensitivity analyses were also conducted to account for three potentially, predictive factors (stage; lymph node dissection; origin) which showed consistent results. Therefore, it seems unlikely our results can solely be related to differences in surgery between Asian and Western countries or the type of lymph node dissection.

Third, for several nodes in both NMAs there were few RCTs available. Therefore, statistical power was lacking for specific comparisons. This might also explain the absent survival benefit of adjuvant chemotherapy in NMA-1 compared to surgery alone contrary to NMA-2 were we found significant benefit for several adjuvant chemo(radio)therapy regimens. Although, the discrepancy could also be related to the amount of R1/R2 resected patients in the adjuvant trials of NMA-1 compared to NMA-2 were all RCTs included R0 resected patients. Another example, is the node taxane-cisplatin in NMA-2 which consisted of 70 patients from one RCT. Moreover, no comparison between the best treatments of both NMAs—such as between taxane-based perioperative chemotherapy and the adjuvant doublet oxaliplatin-fluoropyrimidine—could be made. However, the comparison between taxane-based perioperative chemotherapy and adjuvant chemotherapy in NMA-1 was statistically more robust, due to the fact three out of four adjuvant studies were taxane-based triplet regimens.

Fourth, most RCTs investigating adjuvant therapy compared to observation after curative resection in NMA-2 were conducted between 1990 and 2010. Results must be extrapolated with care to current clinical practice.

## 4. Methods

### 4.1. Protocol

The protocol was registered in PROSPERO, the international prospective register of systematic reviews (CRD42017074888).

### 4.2. Literature Search

Our systematic review was performed in accordance with the Preferred Reporting Items for Systematic Reviews and Meta-Analyses (PRISMA) guidelines [81]. PubMed, EMBASE, and the Cochrane Central Register of Controlled Trials (CENTRAL) were searched for eligible randomized controlled trials up to August 2017. The search strategy consisted of medical subject headings (MeSH) and text words for gastric cancer and esophageal cancer. The search included the term ‘esophageal cancer’ to not miss studies which included both esophageal and gastric cancer patients. Moreover, the meeting abstracts from the American Society of Clinical Oncology (ASCO) (http://ascopubs.org/search/advanced) and European Society for Medical Oncology (ESMO) (https://academic.oup.com/annonc/advanced-search) were searched up to August 2017. The literature search strategy was established and performed by MM and EtV. Three authors (TvdE, RM, FaN) screened the titles, abstracts and full articles independently. All parts of the search were screened by at least two authors in mutual consultation, and disagreements were discussed with a third arbiter (EtV or HvL) until consensus was reached.

### 4.3. Study Selection

Eligibility criteria consisted of the following:

(1) Prospective phase II or III randomized controlled trials.
(2) Patients with pathologically proven gastric adenocarcinoma stage I, II and III (T1–4, N1–3, M0).
(3) The treatment of patients with gastric cancer was with curative intent.
(4) Patients were treated with one or more of the following intravenous or oral cytotoxic agents; fluoropyrimidine (F; either 5-fluorouracil [5-FU], capecitabine [Cap], S-1, tegafur/uracil [UFT], tegafur, or doxifluridine). Platinum-based compounds (cisplatin [C] and oxaliplatin [Ox]). Taxanes (T; either paclitaxel, or docetaxel) or anthracyclines (A; either epirubicin, or doxorubicin). Irinotecan based regimens (I), etoposide (E) and mitomycin C (M) or methotrexate (MTX).
(5) Patients treated with radiotherapy combined with one or more cytotoxic agents (RCh).
(6) Patients treated with targeted agents.

Comparator arm in a randomized controlled trial could consist of chemotherapy (with or without radiotherapy), surgery-alone, irrespective of pathological outcome, (S) or observation after a curative resection (Obs: negative microscopic and macroscopic resection margins; R0). All Studies were included with a D0 or > lymph node dissection. Perioperative and neoadjuvant studies were eligible if they included patients which were deemed resectable with curative intent at inclusion. Trials that solely focused on patients with malignancy of the gastroesophageal junction (GEJ) were excluded, as GEJs are considered esophageal cancer according to the 7th and 8th edition of the American Joint Committee on Cancer (AJCC) [23,82].

### 4.4. Data Extraction and Quality Assessment 

Primary outcome was overall survival (OS). Secondary outcomes were disease free survival (DFS), grade 3 to 4 adverse events (AEs) and complications after surgery (30-day mortality, total morbidity, anastomotic leakage, abdominal abscess, sepsis). Quality of the studies was assessed using the Cochrane Risk of Bias tool (version 5.1.0, The Nordic Cochrane Centre, Denmark). Items were scored as low, high or unknown risk of bias.

### 4.5. Statistical Analysis

Hazard ratios (HR) with 95% confidence intervals (95% CIs) were extracted for OS and DFS to calculate the logHR and standard error based on intention-to-treat study populations. A missing HR was either calculated by the methods described in the paper from Tierney et al. or by digitizing the Kaplan-Meier curves using Plot Digitizer (http://plotdigitizer.sourceforge.net), thereafter the HRs were calculated using the method of Parmar et al. [83,84]. In case neither the HRs nor the Kaplan-Meier curves were provided in the study reports, HRs were extracted from a previously conducted meta-analysis or individual patient data meta-analysis [60,61,62].

For all analyses consisting of at least 10 individual studies, a random effects network meta-analysis (NMA) in the Bayesian framework was conducted, using the GeMTC-standalone version (https://gemtc.drugis.org/signin.html), based on the GeMTC R-package [85]. The model accounted for relative treatment effects in multi-arm trials by manually providing the standard error of the absolute effect in the baseline arm (https://gemtc.drugis.org/manual.html). The number of burn-in iterations was set at 5.000 and the inference iterations at 20,000. To assess a correct posterior distribution, the potential scale reduction factor would be kept below 1.05 and the density plots would provide a smooth regular shape. Run lengths were extended if this was not the case and the Markov chains had not converged [86]. Direct and indirect treatment effects were combined into a single effect size, and the relative effects between all treatments were calculated as combined hazard ratios and 95% credible intervals (95% CrIs). Outcomes were deemed significant at an α-level of 0.05. A point estimate of HRs of 0.80 or less was regarded as clinically relevant [87].

The included randomized controlled trials consisted of different designs. In trials investigating perioperative and neoadjuvant therapy, patients were randomized before undergoing surgery. Most trials investigating adjuvant therapy randomized patients after a curative resection. Thus the patient population of perioperative and neoadjuvant studies is different from adjuvant studies. Therefore, the majority of the trials investigating perioperative and neoadjuvant therapy could not be compared within a single NMA model to adjuvant studies. To be comparable within one NMA, the transitivity assumption has to be fulfilled, which implies that in principle every patient in a study could have been randomized to every treatment in the network [88]. Therefore, we decided to create two networks:

(1) A network comparing different treatment strategies (NMA-1).
(2) A network comparing different adjuvant treatment modalities after curative resection (NMA-2).

Moreover, grade 3–4 toxicity and surgical complications were assessed by pairwise meta-analysis.

### 4.6. Merging of Treatment Groups

The first network (NMA-1) was created to compare treatment strategies as a whole, i.e., perioperative strategies, neoadjuvant strategies and adjuvant strategies. More specifically, in these type of trials in the model, patients were randomized to either perioperative or neoadjuvant therapy before surgery. In addition, studies investigating adjuvant therapy could be included if patients were randomized to this study arm before undergoing surgery and a head-to-head comparison in a randomized controlled trial was available with either perioperative or neoadjuvant treatment.

The following groups were compared: (1) perioperative chemotherapy with non-taxane-containing cytotoxic regimens; (2) perioperative chemotherapy with taxane-containing cytotoxic regimens; (3) perioperative chemotherapy with adjuvant chemoradiotherapy; (4) perioperative chemotherapy with bevacizumab; (5) neoadjuvant chemotherapy and; (6) adjuvant chemotherapy. The merging of different regimens in the strategy network was performed according to the following insights:

(1) In a preliminary network meta-analysis (NMA) without merging of different neoadjuvant, perioperative or adjuvant regimens, there was no significant difference between the separate original treatment regimens.
(2) Taxane-based perioperative chemotherapy was kept separate from standard anthracycline-based perioperative chemotherapy due to statistically significant direct evidence for superiority of taxane-based chemotherapy provided by the recently presented results of the FLOT-4 study [4].
(3) Bevacizumab combined with perioperative chemotherapy (PCB) was kept as a separate node in the network. The ST03 trial did not show any survival benefit in favor of PCB compared to perioperative anthracycline-based chemotherapy [12]. To establish if this is also the case compared to taxane-based perioperative chemotherapy, PCB was analyzed separately from perioperative chemotherapy.

The second model (NMA-2) included RCTs investigating adjuvant therapy for curatively resected gastric cancer. More specifically, studies were included if patients were randomized to adjuvant therapy arms if a curative resection (R0) was achieved.

Cytotoxic agents from the same drug class were taken together based on previous evidence in metastatic esophagogastric cancer [66]. The following drug classes were identified: (1) fluoropyrimidines (F): 5-FU, S-1, capecitabine, UFT, tegafur and doxifluridine (with or without the co-administration of leucovorin [Lv]); (2) anthracyclines (A): epirubicin and doxorubicin; (3) taxanes (T): docetaxel and paclitaxel.

Radiotherapy in combination with one or more cytotoxic agents was grouped together as chemoradiotherapy (RCh). An anthracycline combined with two other cytotoxic agents (two of the following: a fluoropyrimidine, mitomycin C, etoposide or cisplatin) was grouped together as anthracycline-containing triplet (ATr). Oxaliplatin with capecitabine for eight cycles and thereafter eight extra cycles of capecitabine monotherapy was acknowledged as a separate treatment regimen (OxF-prolonged) [50]. Several subanalyses were performed to examine if the merging of drug classes was justified and showed consistency in treatment efficacy, see Supplementary Methods (Section 1.6).

### 4.7. Sensitivity Analysis and Assessment of Inconsistency

Sensitivity analyses were performed for stage: in NMA-1, studies that evaluated patients with stage IV-disease [M1] (discovered during treatment or surgery) were omitted and for NMA-2, studies which only included stage III patients were omitted. To account for potential confounding due to differences in surgical techniques between Asia and Western patients, we conducted two sensitivity analyses for the extend of lymph node dissection (D0/D1 versus D2 or higher) and Asian vs. Western patients. Node-split models were created to assess network consistency (between direct and indirect evidence). In case of inconsistency, baseline characteristics were explored for the corresponding studies. Sensitivity analyses were performed, omitting the studies responsible for network inconsistency one by one.

## 5. Conclusions

Based on currently available data, taxane-containing perioperative chemotherapy is the most promising treatment strategy for resectable gastric adenocarcinoma. If no neoadjuvant treatment has been given, an oxaliplatin-fluoropyrimidine doublet is the most promising adjuvant regimen after a curative resection for resectable gastric adenocarcinoma. The use of adjuvant oxaliplatin has to be further verified in Western gastric cancer patients. Further research is warranted to confirm or reproach our findings.

## Figures and Tables

**Table 1:** Baseline characteristics of studies included in the treatment-strategy network meta-analysis (NMA-1).

Studies	No.	Regimen	Node	Stage ^1^	D2 or > LND No. (%)	Descent	Age, Median, (Range), y	Men No. (%)
Perioperative Chemotherapy vs. Surgery								
Ychou 2011 [11]	113	Peri + Cis + 5-FU	PC	I–IV	D2	W	63 (36-75)	96 (85)
	111	Surg	S	I–IV		W	63 (38–75)	91 (82)
Cunningham 2006 [3]	250	Peri + Epi + Cis + 5-FU	PC	II–III	93 (37)	W	62 (29–85)	205 (82)
	253	Surg	S	II–III	96 (38)	W	62 (23–81)	191 (76)
Perioperative Chemotherapy vs. Perioperative Chemotherapy + Bevacizumab								
Cunningham 2017 [12]	533	Peri + Epi + Cis + Cap	PC	II–III	D1+D2	W	63 (31–79)	434 (82)
	530	Peri + Epi + Cis + Cap + BEV	PCB	II–III		W	64 (28–82)	425 (80)
Perioperative Chemotherapy vs. Perioperative Chemotherapy + Radiotherapy								
Verheij 2016 [13]	393	Peri + Epi + Cis/Ox + Cap	PC	I–III	40 (6)	W	62	264 (67)
	395	Peri + Epi + Cis/Ox + Cap + RT	PCR	I–III		W		265 (67)
Perioperative Chemotherapy vs. Adjuvant Chemotherapy								
Nio 2004 [14]	102	Peri + UFT	PC	I–IV	58 (57)	A	64 (±12)	70 (69)
	193	UFT	AC	I–IV	95 (49)	A	65 (±12)	141 (73)
Perioperative Chemotherapy Taxane Based vs. Perioperative Chemotherapy								
Al-Batran 2017 [4]	356	Peri + Dtx + Ox + 5-FU/Lv	PCT	II–III	D2	W	62	530 (74)
	360	Peri + Epi + Cis + 5-FU/Cap	PC	II–III		W		
Perioperative Chemotherapy Taxane Based vs. Perioperative Chemotherapy + Bevacuzimab								
Ma 2015 [15]	40	Peri + Dtx + Ox + 5-FU/Lv	PCT	II–III	21 (53)	A	53 *	22 (55)
	40	Peri + Dtx + Ox + 5-FU/Lv + BEV	PCB	II–III	31 (78)	A	55 *	24 (60)
Perioperative Chemotherapy Taxane Based vs. Adjuvant Chemotherapy								
Cui 2014 [16]	48	Peri + Ptx + Cis + Tgf	PCT	II–III	NR	A	55 (41–69) *	19 (40)
	48	Ptx + Cis + Tgf	AC	II–III	NR	A	56 (39–72) *	21 (44)
Qu 2010 [17]	39	Peri + Ptx + Ox + 5-FU/Lv	PCT	II–III	NA	A	NA	NA
	39	Ptx + Ox + 5-FU/Lv	AC	II–III	NA	A	NA	NA
Neoadjuvant Chemotherapy vs. Surgery								
Imano 2010 [18]	16	Neo + Cis + 5-FU	NC	II–III	16 (100)	A	58 (±12)	13 (81)
	16	Surg	S	II–III	16 (100)	A	60 (±8)	9 (56)
Schuhmacher 2010 [19]	72	Neo + Cis + 5-FU/Lv	NC	III–IV	67 (96)	W	56 (38–70)	50 (69)
	72	Surg	S	III–IV	63 (93)	W	58 (26–69)	50 (69)
Zhao 2006 [20]	20	Neo + 5-FU/Lv	NC	I–IV	NR	A	58 (32–70) *	NR
	20	Surg	S	I–IV	NR	A		NR
Hartgrink 2004 [21]	29	Neo + Doxo + 5-FU/Lv + MTX	NC	I–IV	D1	W	60 (34–75) *	32 (54)
	30	Surg	S	I–IV		W		
Neoadjuvant Chemotherapy vs. Adjuvant Chemotherapy								
Fazio 2016 [22]	34	Neo + Dtx + Cis + 5-FU	NC	I–IV	62 (90)	W	57 (25–75)	23 (68)
	35	Dtx + Cis + 5-FU	AC	I–IV		W	59 (39–76)	24 (69)

^1^ Staging was done according to the 7th edition of the AJCC and according to the pathological TNM stage [23]. Nio 2004 administered epirubicin, cisplatin and 5-FU to stage IV patients. Qu 2010 and Cui 2014 administered epirubicin, cisplatin and 5-FU after progression. Ma 2015 administered irinotecan, 5-FU and leucovorin when there was no response on initial therapy. * Mean age was given instead of median age. Abbreviations: 5-FU = 5-fluorouracil; A = Asian; AC = adjuvant chemotherapy; BEV = bevacizumab; Cap = capecitabine; Cis = cisplatin; Doxo = doxorubicin; Dtx = docetaxel; Epi = epirubicin; LND = lymph node dissection; Lv = leucovorin; MTX = methotrexate; NA = not available; NC = neoadjuvant chemotherapy; Neo = neoadjuvant; No. = number; NR = not reported; Ox = oxaliplatin; PC = perioperative chemotherapy; PCB = perioperative chemotherapy with bevacizumab; PCT = perioperative taxane-based chemotherapy; PCR = perioperative chemotherapy with adjuvant radiotherapy; Peri = perioperative; Ptx = paclitaxel; S = surgery alone; RT = radiotherapy; Surg = surgery; Tgf = tegafur; UFT = tegafur/uracil; W = western; y = years.

**Table 2:** Baseline characteristics of studies included in the adjuvant therapy for curatively resected gastric cancer network meta-analysis (NMA-2).

Studies	No.	Regimen	Node	Stage ^1^	D2 or > LND No. (%)	Descent	Age, Median, (Range), y	Men No. (%)
Anthracycline + Fluoropyrimidine vs. Observation								
Neri 2001 [24]	69	Epi + 5-FU/Lv	AF	II–III	9 (13)	W	62 (37–73)	50 (72.5)
	68	Observation	Obs	II–III	10 (15)	W	64 (35–74)	48 (70.6)
Krook 1991 [25]	61	Doxo + 5-FU	AF	I–III	NR	W	63 (33–77)	47 (77)
	64	Observation	Obs	I–III	NR	W	62 (38–78)	51 (80)
Anthracycline + Doublet vs. Observation								
Kulig 2010 [26]	141	Doxo + Eto + Cis	ATr	I–III	112 (79)	W	61 (58–67)	100 (71)
	154	Observation	Obs	I–III	123 (80)	W	64 (61–66)	111 (72)
Di Costanzo 2008 [27]	130	Epi + Cis + 5-FU/Lv	ATr	I–III	71 (55)	W	59	79 (61)
	128	Observation	Obs	I–III	72 (56)	W	59	78 (61)
De Vita 2007 [28]	112	Epi + Eto + 5-FU/Lv	ATr	I–III	0	W	63 (39–70)	66 (59)
	113	Observation	Obs	I–III	0	W	62 (41–70)	65 (58)
Tentes 2006 [29]	20	Doxo + MMC + 5-FU	ATr	II–III	20 (100)	W	65 (±10) *	14 (70)
	20	Observation	Obs	II–III	20 (100)	W	65 (±11) *	11 (55)
Tsavaris 1996 [30]	42	Epi + MMC + 5-FU	ATr	III	NR	W	53 (41–65) *	32 (76)
	42	Observation	Obs	III	NR	W	57 (35–66) *	25 (60)
Lise 1995 [31]	155	Doxo + MMC + 5-FU	ATr	II–III	84 (27)	W	<71 years	94 (61)
	159	Observation	Obs	II–III		W	<71 years	108 (68)
Coombes 1990 [32]	133	Doxo + MMC + 5-FU	ATr	II–III	NR	W	57 *	93 (70)
	148	Observation	Obs	II–III	NR	W	57 *	98 (68)
Anthracycline + Etoposide + Cisplatin + Fluoropyrimidine vs. Observation								
Bajetta 2002 [33]	135	Doxo + Eto + Cis + 5-FU/Lv	AECF	II–III	Maj.	W	57 (23–70)	81 (59)
	136	Observation	Obs	II–III	Maj.	W	57 (31–70)	93 (68)
Anthracycline + Doublet vs. Fluoropyrimidine								
Cascinu 2007 [34]	201	Epi + Cis + 5-FU/Lv	ATr	II–III	312 (79)	W	58	135 (67)
	196	5FU/Lv	F	II–III		W	59	120 (61)
Lee 2004 [35]	32	Epi + Cis + 5-FU/Lv	ATr	III	32 (100)	A	53 (31–61)	13 (41)
	29	5-FU	F	III	29 (100)	A	52 (26–66)	13 (45)
Anthracycline + Fluoropyrimidine vs. Mitomycin C + Fluoropyrimidine vs. Fluoropyrimidine								
Tsujinaka 2000 [36]	61	Epi + 5-FU	AF	I–II	60 (98)	A	≤75 years	38 (62)
	62	MMC + 5-FU	MF	I–II	61 (98)	A	≤75 years	44 (71)
	62	5-FU	F	I–II	61 (98)	A	≤75 years	44 (71)
Anthracycline + Doublet vs. Mitomycin C + Fluoropyrimidine vs. Fluoropyrimidine								
Chang 2002 [37]	131	Doxo + MMC + 5-FU	ATr	I–III	131 (100)	A	51 (26–70)	100 (76)
	131	MMC + 5-FU	MF	I–III	131 (100)	A	54 (23–74)	96 (73)
	133	5-FU	F	I–III	133 (100)	A	53 (21–75)	99 (74)
Cisplatin + Fluoropyrimidine vs. Observation								
Bouche 2005 [38]	127	Cis + 5-FU	CF	II–III	70 (27)	W	60 (32–82)	93 (73)
	133	Observation	Obs	II–III		W	62 (31–83)	93 (70)
Chipponi 2004 [39]	93	Cis + 5-FU/Lv	CF	II–III	D1+D2	W	59 *	58 (62)
	103	Observation	Obs	II–III		W	63 *	71 (69)
Fluoropyrimidine vs. Observation								
Sasako 2011 [40]	529	S-1	F	II–III	529 (100)	A	63 (27–80)	367 (69)
	530	Observation	Obs	II–III	530 (100)	A	63 (33–80)	369 (70)
Nakajima 2007 [41]	93	UFT	F	II–III	93 (100)	A	63	75 (70)
	95	Observation	Obs	II–III	95 (100)	A	64	77 (73)
Mitomycin C vs. Observation								
Grau 1993 [42]	68	MMC	M	I–III	NR	W	56 *	44 (65)
	66	Observation	Obs	I–III	NR	W	57 *	44 (67)
Mitomycin C + Fluoropyrimidine vs. Observation								
Cirera 1999 [43]	76	MMC + Tgf	MF	I–III	76 (100)	W	61 *	52 (68)
	72	Observation	Obs	I–III	72 (100)	W	61 *	42 (58)
Kim 1992 [44]	77	MMC + 5-FU	MF	III	77 (100)	A	(30–70)	NR
	94	Observation	Obs	III	94 (100)	A	(30–70)	NR
Mitomycin C + Fluoropyrimidine vs. Mitomycin C								
Grau 1998 [45]	40	MMC + Tgf	MF	I–III	D1+D2	W	62 (36–75)	27 (68)
	45	MMC	M	I–III		W	63 (22–75)	27 (60)
Mitomycin C + Cisplatin + Fluoropyrimidine vs. Mitomycin C + Fluoropyrimidine								
Kang 2013 [46]	431	MMC + Cis + 5DFUR	MCF	II–III	431 (100)	A	55 (20–70)	294 (68)
	424	MMC + 5DFUR	MF	II–III	424 (100)	A	56 (29–70)	294 (69)
Mitomycin C + Cisplatin + Fluoropyrimidine vs. Cisplatin + Fluoropyrimidine								
Shimoyama 1999 [47]	12	MMC + Cis + UFT (600 mg)	MCF	I–III	D1+D2	A	65 (±8)	13 (77)
	17	Cis + UFT	CF	I–III		A	64 (±8)	8 (67)
Oxaliplatin + Fluoropyrimidine vs. Observation								
Noh 2014 [48]	520	Ox + Cap	OxF	II–III	520 (100)	A	56 (±11) *	373 (72)
	515	Observation	Obs	II–III	515 (100)	A	56 (±11) *	358 (70)
Oxaliplatin + Fluoropyrimidine vs. Fluoropyrimidine								
Zhang 2011 [49]	42	Ox + 5-FU/Lv	OxF	II–III	42 (100)	A	48	25 (60)
	38	5-FU/Lv	F	II–III	38 (100)	A	54	24 (63)
Oxaliplatin + Fluoropyrimidine Prolonged vs. Oxaliplatin + Fluoropyrimidine								
Feng 2015 [50]	152	Ox + Cap (Prolonged)	OxFPr	II–III	152 (100)	A	61 (±11)	104 (67)
	155	Ox + Cap	OxF	II–III	155 (100)	A	60 (±10)	99 (65)
Radiotherapy + Chemotherapy vs. Observation								
Smalley 2012 [6]	281	RT + 5-FU/Lv	RCh	I–III	54 (10)	W	60 (25–87)	202 (72)
	275	Observation	Obs	I–III		W	59 (23–80)	195 (71)
Radiotherapy + Chemotherapy vs. Fluoropyrimidine								
Kim 2012 [51]	46	RT + 5-FU/Lv	RCh	III	46 (100)	A	9> 60	34 (74)
	44	5-FU/Lv	F	III	44 (100)	A	14>60	25 (57)
Yu 2012 [52]	34	RT + 5-FU/Lv	RCh	II–III	D1+D2	A	NR	NR
	34	5-FU/Lv	F	II–III		A	NR	NR
Zhu 2012 [53]	186	RT + 5-FU/Lv	RCh	I–III	205 (100)	A	56 (38–73)	135 (73)
	165	5-FU/Lv	F	I–III	175 (100)	A	59 (42–75)	126 (76)
Radiotherapy + Chemotherapy vs. Cisplatin + Fluoropyrimidine								
Park 2015 [54]	230	RT + Cis + Cap	RCh	I–III	230 (100)	A	56 (28–76)	143 (62)
	228	Cis + Cap	CF	I–III	228 (100)	A	56 (22–77)	153 (67)
Kwon 2010 [55]	31	RT + Cis + Cap + 5-FU	RCh	III	31 (100)	A	8 ≥ 60	21 (68)
	30	Cis + 5-FU	CF	III	30 (100)	A	14 ≥ 60	23 (77)
Radiotherapy + Chemotherapy vs. Taxane + Cisplatin								
Bamias 2010 [56]	72	RT + Dtx + Cis/Car	RCh	II–III	D0+D1+D2	W	63 (32–75)	48 (67)
	71	Dtx + Cis/Car	TC	II–III		W	62 (41–79)	52 (73)
Taxane + Fluoropyrimidine vs. Cisplatin + Fluoropyrimidine								
Lee 2016 [57]	75	Dtx + S-1	TF	III	75 (100)	A	NR	NR
	78	Cis + S-1	CF	III	78 (100)	A	NR	NR
Taxane + Irinotecan + Cisplatin + Fluoropyrimidine vs. Fluoropyrimidine or Mitomycin C								
Bajetta 2014 [58]	562	Dtx + IRI + Cis + 5-FU/Lv	TICF	II–III	796 (72)	W	≤75 years	NR
	538	5-FU/Lv	F	II–III		W	≤75 years	NR
Di Bartolomeo 2006 [59]	85	Dtx + IRI + Cis + 5-FU/Lv	TICF	II–III	66 (77)	W	10 ≥ 70	60 (71)
	81	MMC	M	II–III	62 (76)	W	8 ≥ 70	55 (68)

^1^ Staging was done according to the 7th edition of the AJCC and according to the pathological TNM stage [23]. * Mean age was given instead of median age. Abbreviations: 5-DFUR = doxifluridine; 5-FU = 5-fluorouracil; A = anthracycline; A descent = Asian; ATr = anthracycline-based triplet; Cap = capecitabine; Car = carboplatin; C = cisplatin; Cis = cisplatin; Doxo = doxorubicin; Dtx = docetaxel; E = etoposide; Epi = epirubicin; Eto = etoposide; F = fluoropyrimidine; I = irinotecan; IRI = irinotecan; LND = lymph node dissection; Lv = leucovorin; M = mitomycin C; MMC = mitomycin C; No. = number; NR = not reported; Obs = observation; Ox = oxaliplatin; OxFpr = doublet oxaliplatin with an one year treatment with a fluoropyrimidine; RT = radiotherapy; RCh = chemoradiotherapy; T = taxane; Tgf = tegafur; UFT = uracil/tegafur; W = western; y = years.

## Supplementary Materials

The following are available online at http://www.mdpi.com/2072-6694/11/1/80/s1, Supplementary Methods and Results. Figure S1: Results for NMA-1 when neoadjuvant taxane and adjuvant taxane containing chemotherapy were separated from non-taxane containing neoadjuvant or adjuvant chemotherapy compared to surgery alone, Figure S2: Results for NMA-1 when neoadjuvant taxane and adjuvant taxane containing chemotherapy were separated from non-taxane containing neoadjuvant or adjuvant chemotherapy, Figure S3: Overall survival pair wise meta-analysis based on treatment strategy, Figure S4: Overall survival pair wise meta-analysis of adjuvant therapy for curatively resected gastric cancer, Figure S5: Disease free survival pair wise meta-analysis of adjuvant therapy for curatively resected gastric cancer, Figure S6: Overall survival risk of bias assessment for the treatment strategy NMA-1, Figure S7: Overall survival and disease free survival risk of bias assessment for the adjuvant therapy after curative resection NMA-2, Figure S8: Direct and combined hazard ratios for overall survival in the treatment strategy NMA-1, Figure S9: Direct and combined hazard ratios for overall survival in the adjuvant therapy after curative resection NMA-2, Figure S10: Direct and combined hazard ratios for disease free survival in the adjuvant therapy after curative resection NMA-2, Table S1: Grade 3-4 toxicity for the treatment strategy NMA-1, Table S2: Surgical mortality and morbidity for the treatment strategy NMA-1, Table S3: Grade 3-4 toxicity for the adjuvant therapy after curative resection NMA-2.
