# Supplementary material for: The Efficacy and Safety of (Neo)Adjuvant Therapy for Gastric Cancer: A Network Meta-analysis
Source: Cancers (Basel). 2019 Jan 11;11(1):80. doi: 10.3390/cancers11010080 (PMC6356558; doi:10.3390/cancers11010080)
Supplement: Supplementary file 1 [file cancers-11-00080-s001.zip › cancers-407394- supplementary materials/cancers-407394 - Supple.-final check.docx]

Supplementary Materials: The Efficacy and Safety of (Neo)Adjuvant Therapy for Gastric Cancer: A Network Meta-analysis

Tom van den Ende, Emil ter Veer, Mélanie Machiels, Rosa M. A. Mali, Frank A. Abe Nijenhuis, Laura de Waal, Marety Laarman, Suzanne S. Gisbertz, Maarten C. C. M. Hulshof, Martijn G. H. van Oijen and Hanneke W. M. van Laarhoven

1. Supplementary Methods

1.1. Cochrane Central Register of Controlled Trials (CENTRAL)

| **#** | **Searches** |
| --- | --- |
| 1 | MeSH descriptor: [Esophageal Neoplasms] explode all trees |
| 2 | MeSH descriptor: [Stomach Neoplasms] explode all trees |
| 3 | (gastric or stomach or esophagus or oesophagus or esophageal or oesophageal) near (neoplasm * or cancer * or carcinoma or adenocarcino * or tumor * or malign * neoplasm * neoplasm *): ti, ab, kw (Word variations have been searched) |
| 4 | #1 or #2 or #3 |
| 5 | (chemotherapy or radiotherapy or chemoradiotherapy or radiation or radiochemotherapy) near/1 (adjuvant or neoadjuvant or neo-adjuvant or combined or perioperative or peri-operative or preoperative or pre-operative or postoperative or post-operative): ti, ab, kw (Word variations have been searched) |
| 6 | MeSH descriptor: [Gastrectomy] explode all trees |
| 7 | MeSH descriptor: [Esophagectomy] explode all trees |
| 8 | MeSH descriptor: [Chemotherapy, Adjuvant] explode all trees |
| 9 | MeSH descriptor: [Radiotherapy, Adjuvant] explode all trees |
| 10 | MeSH descriptor: [Chemoradiotherapy] explode all trees |
| 11 | MeSH descriptor: [Radiotherapy] explode all trees |
| 12 | #5 or #6 or #7 or #8 or #9 or #10 or #11 |
| 13 | MeSH descriptor: [Treatment Outcome] explode all trees |
| 14 | MeSH descriptor: [Disease-Free Survival] explode all trees |
| 15 | MeSH descriptor: [Mortality] explode all trees |
| 16 | survival or safe * or mortality or quality of life or QOL: ti, ab, kw (Word variations have been searched) |
| 17 | MeSH descriptor: [Quality of Life] explode all trees |
| 18 | #13 or #14 or #15 or #16 or #17 |
| 19 | #4 and #12 and #18 in Trials |

1.2. EMBASE via Ovid

| **#** | **Searches** |
| --- | --- |
| 1 | exp * esophagus tumor/ or exp * stomach tumor/ or ((gastric or stomach or esophagus or oesophagus or esophageal or oesophageal) adj (neoplasm * or cancer * or carcinoma or adenocarcino * or tumor * or malign * neoplasm * neoplasm *)). ti, ab, kw. |
| 2 | gastrectomy/ or * esophagus resection/ or * lymph node dissection/ or exp cancer adjuvant therapy/ or adjuvant chemoradiotherapy/ or cancer radiotherapy/ or gastrectomy. ti, ab, kw. or (exp chemoradiotherapy/ and adjuvant therapy/) or ((chemotherapy or radiotherapy or chemoradiotherapy or radiation or radiochemotherapy) adj1 (adjuvant or neoadjuvant or perioperative or peri-operative or preoperative or pre-operative or postoperative or post-operative)). ti, ab, kw. |
| 3 | controlled clinical trial/ or randomized controlled trial/ or “clinical trial (topic)”/ or (randomized or randomised or randomly). ti, ab, kw. or (trial or effecti *). ti. |
| 4 | exp treatment outcome/ or exp “quality of life”/ or disease-free survival/ or exp mortality/ or (survival or safe * or mortality or quality of life or QOL). ti, ab, kw. |
| 5 | 1 and 2 and 3 |
| 6 | “review”/ not “clinical trial (topic)”/ |
| 7 | lung. ti. |
| 8 | (1 and 2 and 3 and 4) not 6 not 7 |
| 9 | limit 8 to (dutch or english) |

1.3. Medline via PubMed

| **#** | **Searches** |
| --- | --- |
| 1 | esophageal neoplasms/ or stomach neoplasms/ or ((gastric or stomach or esophagus or oesophagus or esophageal or oesophageal) adj (neoplasm * or cancer * or carcinoma or adenocarcino * or tumor * or malign * neoplasm * neoplasm *)). ti, ab, kw. |
| 2 | exp Gastrectomy/ or Esophagectomy/ or Lymph Node Excision/ or Chemotherapy, Adjuvant/ or Radiotherapy, Adjuvant/ or gastrectomy.ti,ab,kw. or ((exp Chemoradiotherapy/ or exp Radiotherapy/) and (Neoadjuvant Therapy/ or adjuvant or neoadjuvant or neo-adjuvant). ti, ab, kw.) or ((chemotherapy or radiotherapy or chemoradiotherapy or radiation or radiochemotherapy) adj1 (adjuvant or neoadjuvant or neo-adjuvant or combined or perioperative or peri-operative or preoperative or pre-operative or postoperative or post-operative)). ti, ab, kw. |
| 3 | exp treatment outcome/ or “Quality of Life”/ or disease-free survival/ or exp Mortality/ or (survival or safe* or mortality or quality of life or QOL). ti, ab, kw. |
| 4 | controlled clinical trial/ or randomized controlled trial/ or Clinical Trials as Topic/ or (randomized or randomly). ti, ab, kw. or (trial or effecti *). ti. |
| 5 | 1 and 2 and 3 and 4 |
| 6 | “review”/ not Clinical Trials as Topic/ |
| 7 | lung.ti. |
| 8 | (1 and 2 and 3 and 4) not 6 not 7 |
| 9` | limit 8 to (dutch or english) |

1.4. Conference Search: American Society of Clinical Oncology

Searching journal content for gastric (all words) in title or abstract and random* in full text, from earliest publication date through August 2017.

1.5. Conference Search: European Society of Medical Oncology

Searching journal content for gastric (all words) in title or abstract and random* in full text, from earliest publication date through August 2017.

1.6. Subanalyses before Merging of Cytotoxic Agents in NMA-2

(1) The fluoropyrimidines:

- In a previously conducted direct comparison adjuvant S-1 was superior to UFT in the SAMIT trial [1]. However, inclusion criteria of the SAMIT trial were T4a, T4b tumors and both R0 and R1 resected patients, results could therefore not be extrapolated to our NMA. Through indirect comparison (preliminary NMA) similar efficacy was noted and UFT/S-1 were grouped together.
- In a previously conducted fluoropyrimidine NMA in the metastatic setting no difference was observed between S-1, capecitabine and 5-FU [2].
- Indirect comparison confirmed similar efficacy between the fluoropyrimidines with equal backbones in a preliminary performed NMA (fluoropyrimidines were not merged together).

(2) The anthracyclines:

- In an indirect comparison with equal backbone similar efficacy was noted between epirubicin and doxorubicin [3].
- Indirect comparison (preliminary NMA) between epirubicin+5-FU [4,5] and doxorubicin+5-FU [6] revealed a significant difference in terms of efficacy in favour of epirubicin, however considering all the indirect evidence and similar efficacy between anthracyclines in triplet combinations (ATr) after indirect comparison (preliminary NMA), epirubicin and doxorubicin were grouped together.

(3). The taxanes:

- No direct or indirect comparison for paclitaxel or docetaxel was available; based on previous direct [7] and indirect [8] evidence both taxanes were grouped together.

(4). Chemoradiotherapy:

- Direct comparison between epirubicin+cisplatin+5-FU+radiotherapy and 5-FU+radiotherapy revealed similar efficacy [9]; indirect comparison also revealed no significant difference between the different backbone schemes of chemoradiotherapy.

2. Supplementary Results

2.1. OS Results for the Strategy NMA-1 When Neoadjuvant Taxane and Adjuvant Taxane Containing Chemotherapy Were Seperated from Non-Taxane Containing Neoadjuvant or Adjuvant Chemotherapy


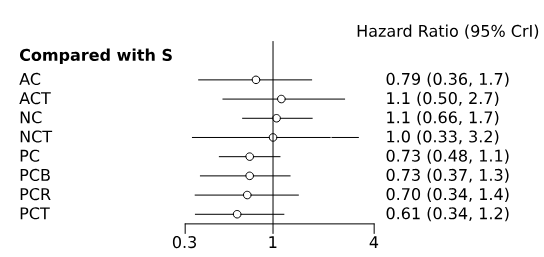


**Figure S1.** Results for NMA-1 when neoadjuvant taxane and adjuvant taxane containing chemotherapy were separated from non-taxane containing neoadjuvant or adjuvant chemotherapy compared to surgery alone. Abbreviations: AC = adjuvant chemotherapy without a taxane; ACT = adjuvant chemotherapy with a taxane; NC = neoadjuvant chemotherapy without a taxane; NCT = neoadjuvant chemotherapy with a taxane; PC = perioperative chemotherapy without a taxane; PCB = perioperative chemotherapy combined with bevacizumab; PCR = perioperative chemotherapy combined with adjuvant chemoradiotherapy; PCT = taxane-based perioperative chemotherapy; S = surgery only.

**Figure S2.** Results for NMA-1 when neoadjuvant taxane and adjuvant taxane containing chemotherapy were separated from non-taxane containing neoadjuvant or adjuvant chemotherapy. The hazard ratio for a given comparison could be read in the intersection of two treatments. The strategies are grouped according to their baseline efficacy compared with surgery-alone. Abbreviations: AC = adjuvant chemotherapy without a taxane; ACT = adjuvant chemotherapy with a taxane; NC = neoadjuvant chemotherapy without a taxane; NCT = neoadjuvant chemotherapy with a taxane; PC = perioperative chemotherapy without a taxane; PCB = perioperative chemotherapy combined with bevacizumab; PCR = perioperative chemotherapy combined with adjuvant chemoradiotherapy; PCT = taxane-based perioperative chemotherapy; S = surgery only.

2.2. Baseline Characteristics NMA-1

The median age was 62 (range = 23–85) years of the patients in the treatment strategy NMA-1. Gender and performance status were roughly balanced between the studies. Additional characteristics of interest included: disease stage at inclusion I-III, D2 or > lymph node dissection and Asian/Western origin (Table 1). Stage at inclusion in the RCTs ranged between I–III, D2 lymph node dissection was not always performed both in Western and Asian studies (Table 1). There were six Asian studies (*n* = 621 patients) and eight Western studies (*n* = 3566 patients). The results of the pair wise meta-analyses for both NMA-1 and NMA-2 can be found below (Figure S3–S5). Risk of bias assessment for OS in the NMA-1 comparing treatment strategies is summarized in Figure S6. Four (29%) studies were rated as low risk of bias. The number of studies that were rated as unclear risk of bias on one or on two items were respectively four (29%) and two (14%). Four studies (29%) were rated unclear risk of bias on three or more items.

2.3. Baseline Characteristics NMA-2

The median age was 59 (range = 20–87) years of the patients in the NMA-2 comparing adjuvant regimens after curative resection. Gender and performance status were roughly balanced between studies (Table 2). D2 or > lymph node dissection was more frequently performed in studies which included Asian patients than in studies with Western patients (Table 2). There were 17 studies including Asian patients (*n* = 5546) and 20 studies including Western patients (*n* = 5215). Risk of bias assessment for OS in the NMA-2 comparing adjuvant therapy after a curative resection is summarized below (Figure S7 A). For OS, 17 (46%) studies were rated as low risk of bias. The number of studies which were rated as unclear risk of bias on one, on two items, or on three or more items were seven (19%), nine (24%), and four (11%). The risk of bias assessment for DFS was summarized in Figure S7 B.

2.4. Network Consistency and Sensitivity Analyses

The treatment strategy NMA-1 consisted of 9 direct comparisons for OS with eight loops for the assessment of (in)consistency. The NMA-2 for curatively resected gastric cancer consisted of 25 direct comparisons for OS and 22 for DFS. In total there were 21 loops for OS and 15 loops for DFS to assess (in)consistency. Node-split models were non-significant for both NMAs. For exploratory purposes, both direct and combined hazard ratios are shown below.

In the NMA-1, three sensitivity analyses were conducted by omitting studies with stage IV (M1) patients detected after inclusion, studies in which patients received a D0/D1 dissection and studies which only included Asian patients. There were not enough studies to perform an NMA with only Asian patients.

For the NMA-2: when all studies with stage III patients only were removed, the results remained largely unaffected for OS and DFS. When all studies with a D2 or > lymph node dissection were omitted OxFpr, OxF and TF were not available for OS, and MCF for DFS, the rest of the results remained robustly the same. Results remained roughly unchanged after omitting all studies with a D0/D1 dissection. After omitting all Asian patients no comparison was available for OxFpr, OxF, MCF and TF. Only the hazard ratio for MF compared to observation alone changed considerably from: 0.75 (CrI 0.59–0.95) to 0.58 (CrI 0.37–0.91) for OS, the same was observed for DFS. However, the remaining comparisons in the analysis remained relative robust both for OS and DFS.


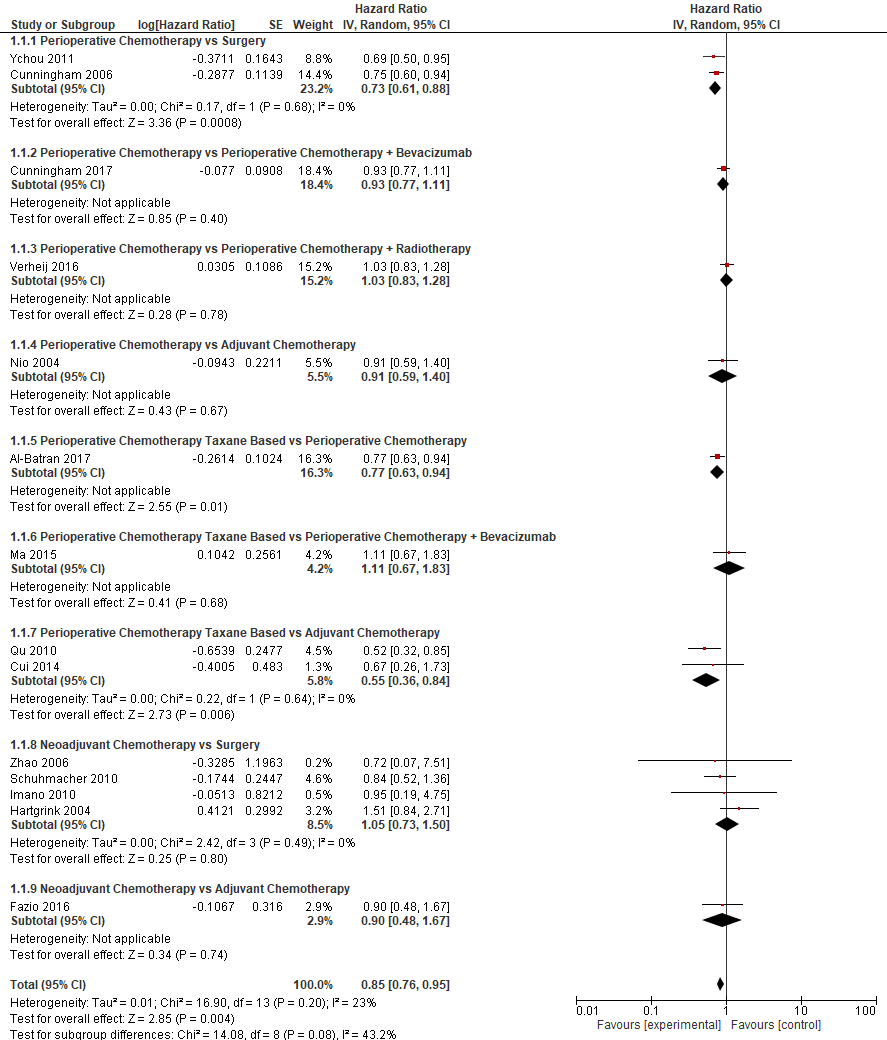


**Figure S3.** Overall survival pair wise meta-analysis based on treatment strategy.


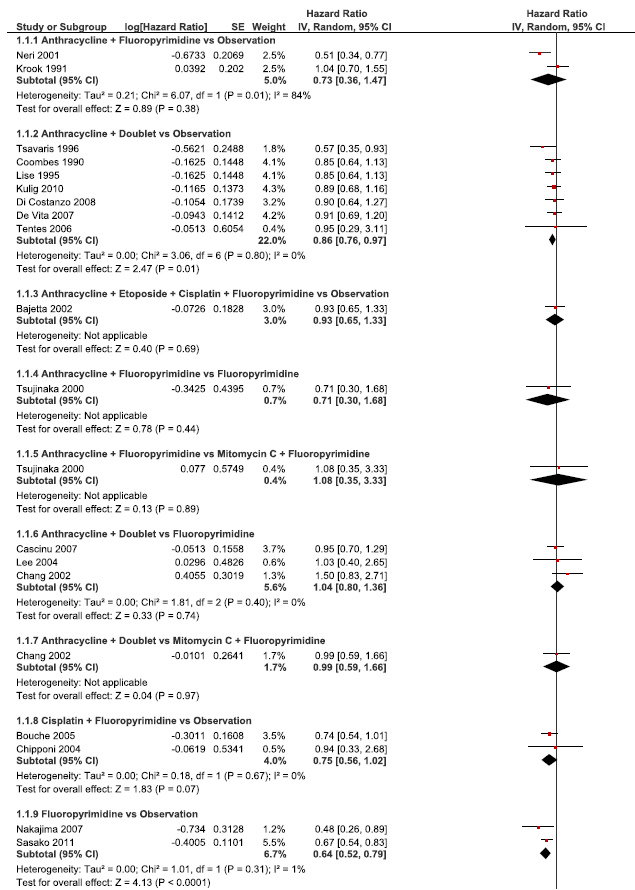
**
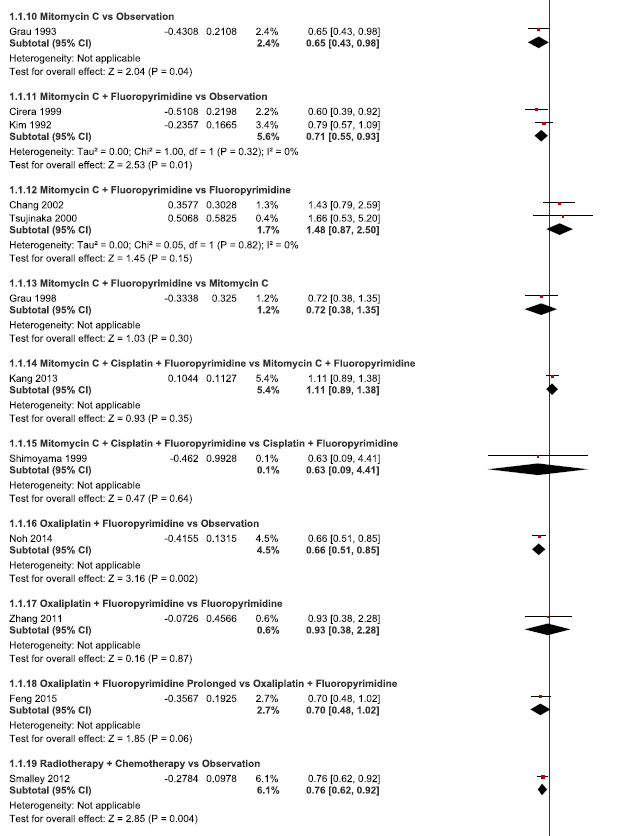

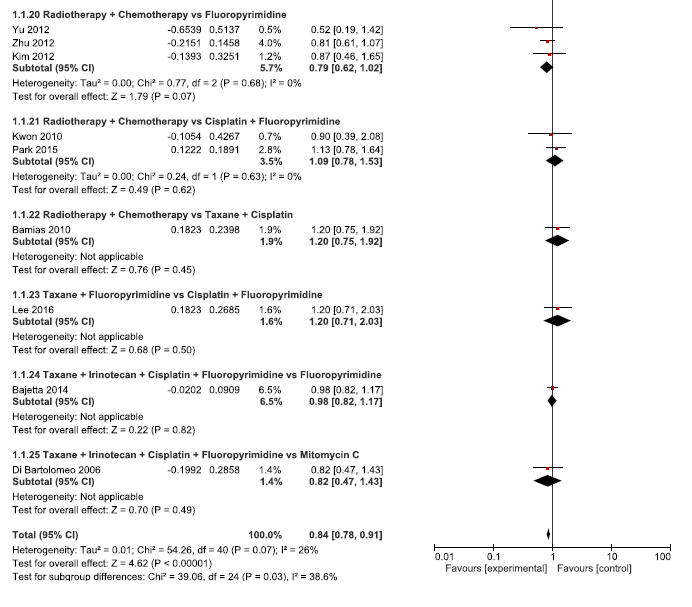
**

**Figure S4.** Overall survival pair wise meta-analysis of adjuvant therapy for curatively resected gastric cancer.


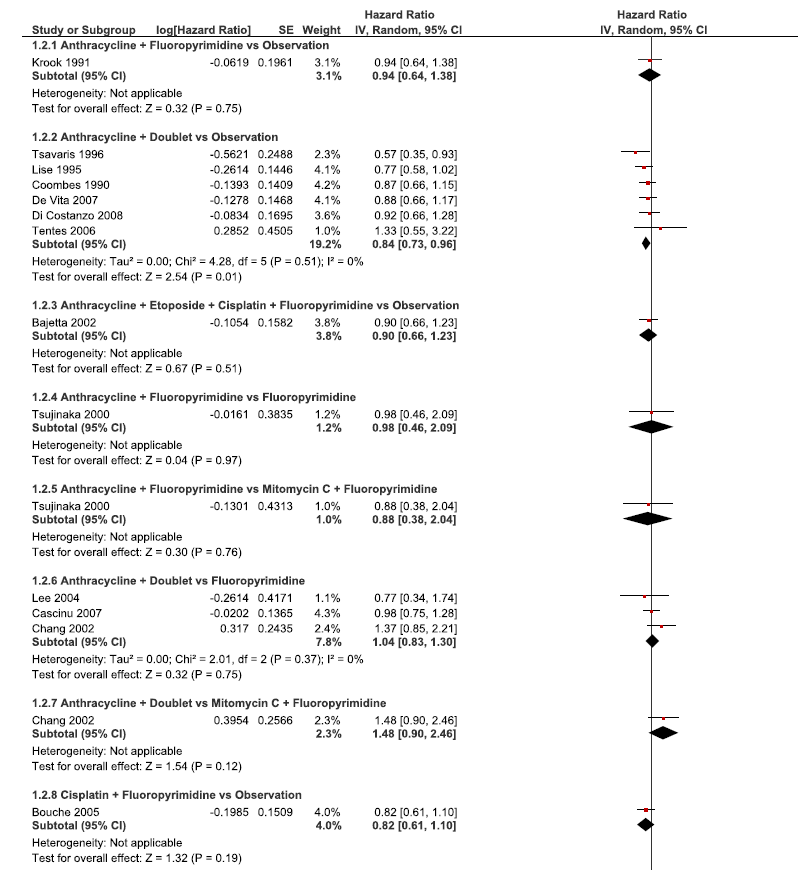


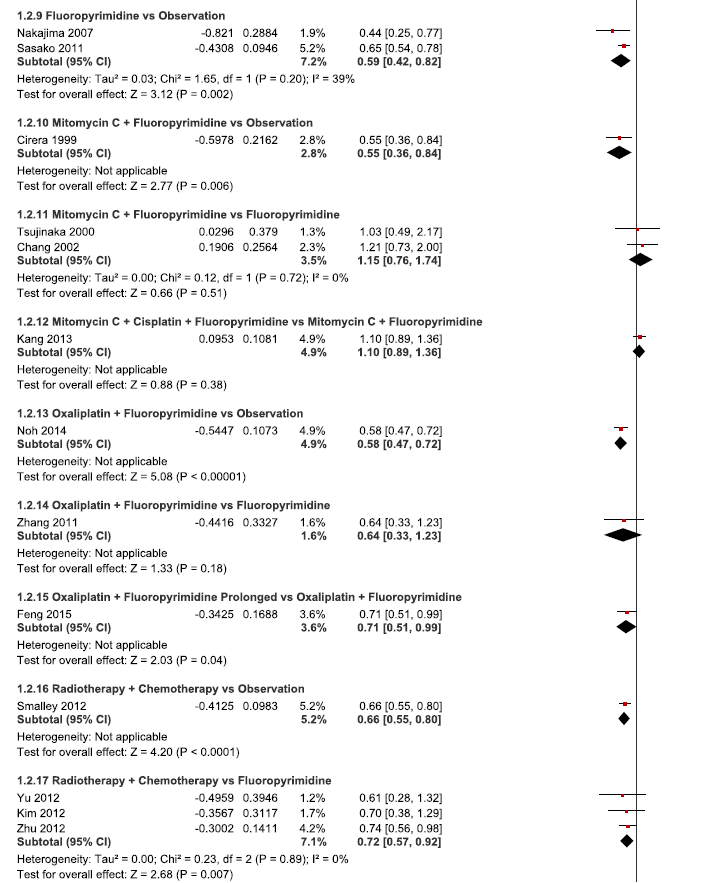


**
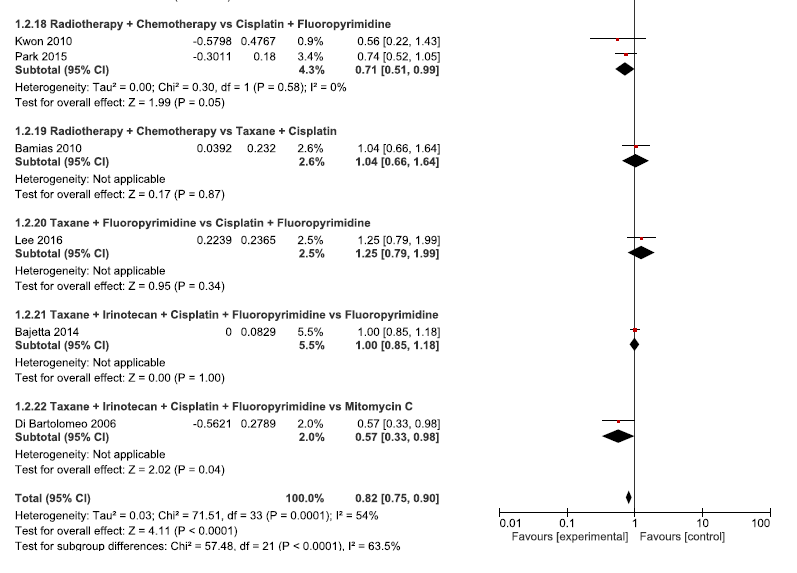
****Figure S5.** Disease free survival pair wise meta-analysis of adjuvant therapy for curatively resected gastric cancer.


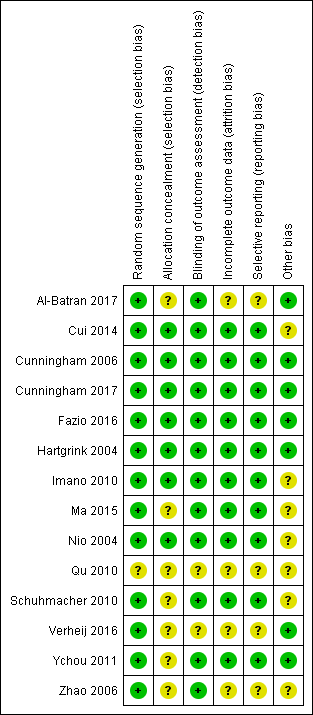


**Figure S6.** Overall survival risk of bias assessment for the treatment strategy NMA-1. Single-centre studies were scored as unknown risk of bias on the item ‘other biases’. The absence of a description of a blinded imaging review committee was not regarded as bias for OS, since the primary outcome OS would not be influenced by this parameter. + = Low risk of bias; ? = unknown risk of bias.


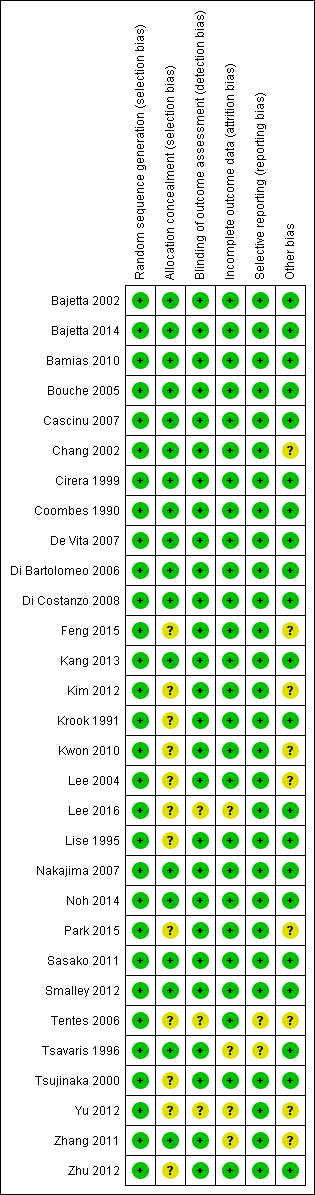

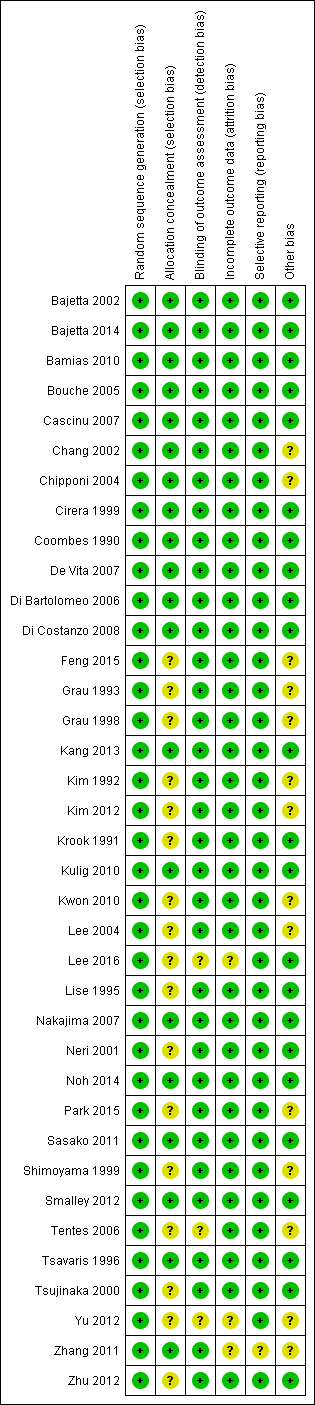


**B**

**A**

**Figure S7.** Overall survival and disease free survival risk of bias assessment for the adjuvant therapy after curative resection NMA-2. Single-centre studies were scored as unknown risk of bias on the item ‘other biases’. The absence of a description of a blinded imaging review committee was not regarded as bias for OS, since the primary outcome OS would not be influenced by this parameter. + = Low risk of bias; ? = unknown risk of bias. (**A**) overall survival; (**B*)*** disease free survival.


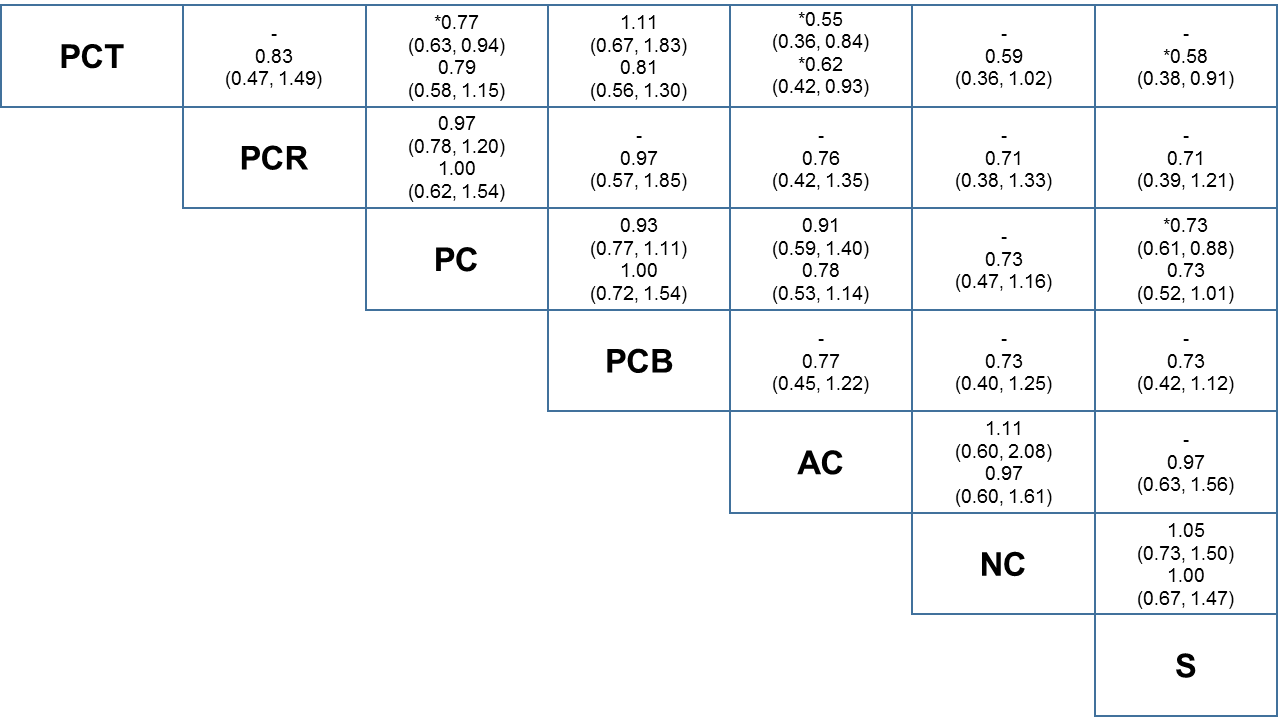


**Figure S8.** Direct and combined hazard ratios for overall survival in the treatment strategy NMA-1. Relative effects in both direct and combined hazard ratios (HR) and 95% Credible Intervals (95% CrI) are shown to observe possible discrepancies between evidence derived from direct comparisons (top of each cell) and evidence derived from both direct and indirect comparisons (bottom of each cell). All *z*-tests to compare two treatments were performed two-sided. * *p* < 0.05. Abbreviations: AC = adjuvant chemotherapy; NC = neoadjuvant chemotherapy; PC = perioperative chemotherapy regimens without a taxane; PCB = perioperative chemotherapy combined with bevacizumab; PCR = perioperative chemotherapy combined with adjuvant chemoradiotherapy; PCT = taxane-based perioperative chemotherapy; S= surgery only.

**
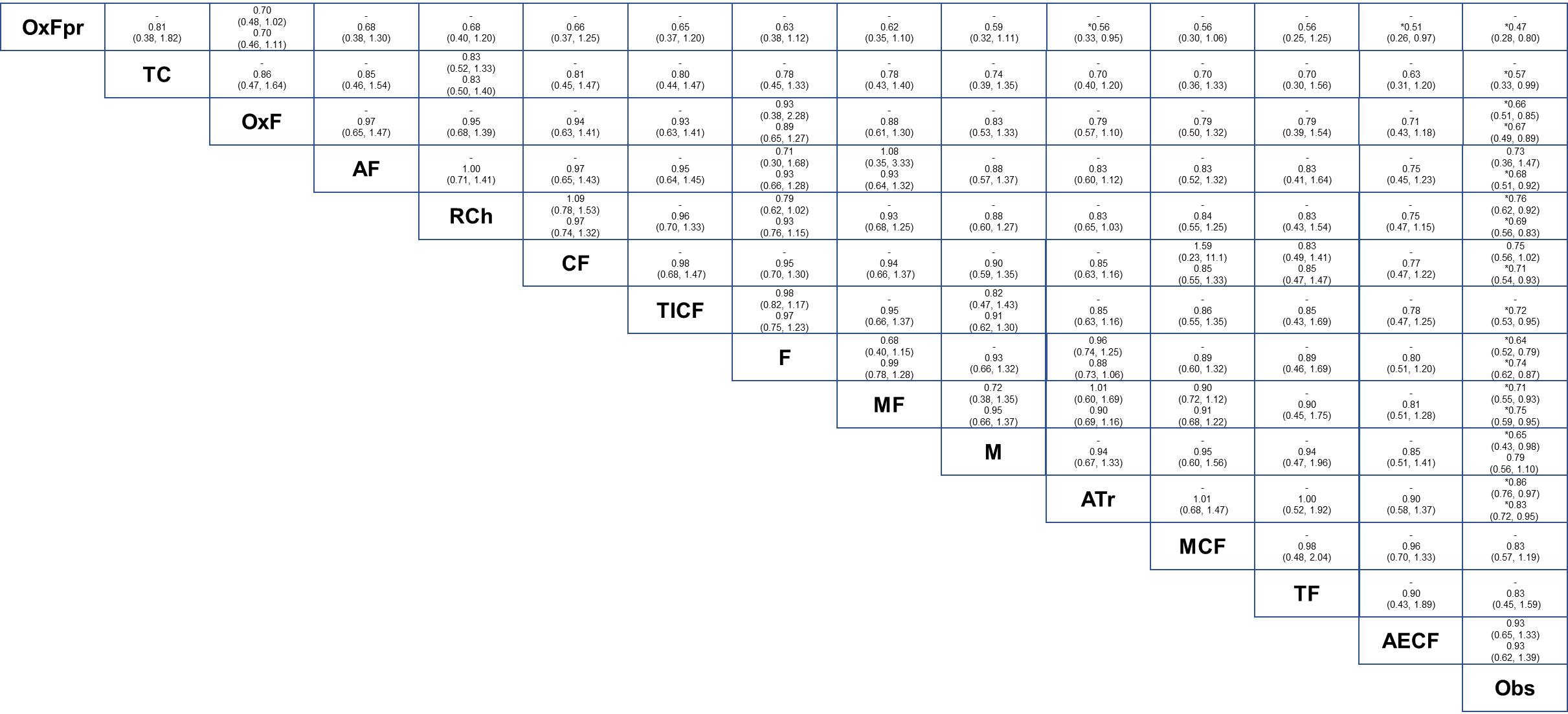
**

**Figure S9.** Direct and combined hazard ratios for overall survival in the adjuvant therapy after curative resection NMA-2. Relative effects in both direct and combined hazard ratios (HR) and 95% Credible Intervals (95% CrI) are shown to observe possible discrepancies between evidence derived from direct comparisons (top of each cell) and evidence derived from both direct and indirect comparisons (bottom of each cell). All *z*-tests to compare two treatments were performed two-sided. * *p* < 0.05. Abbreviations: A = anthracycline; ATr = anthracycline-based triplet; C = cisplatin; E = etoposide; F = fluoropyrimidine; I = irinotecan; M = mitomycin C; Obs = observation; Ox = oxaliplatin; OxFpr= doublet oxaliplatin with an one year treatment with a fluoropyrimidine; RCh = chemoradiotherapy; T = taxane.

**
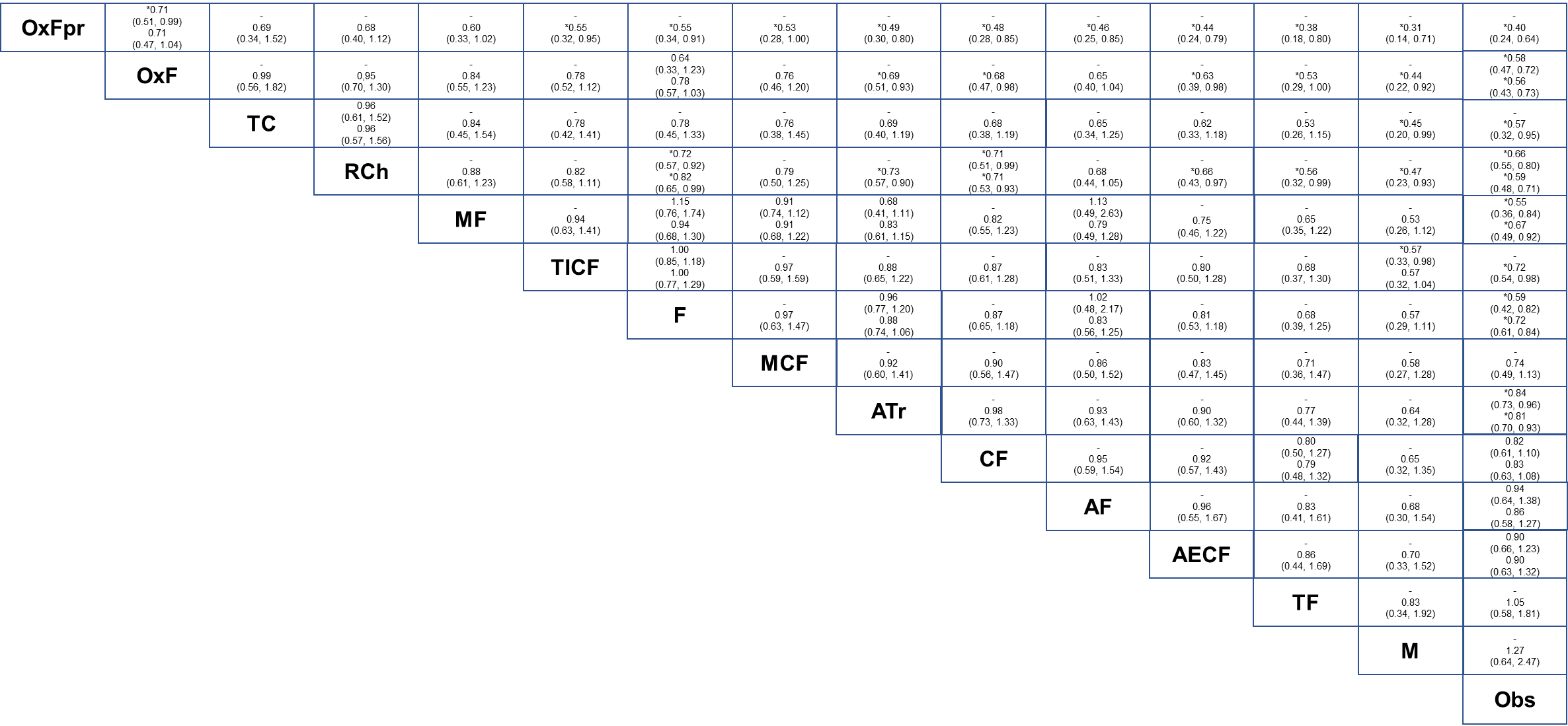
**

**Figure S10.** Direct and combined hazard ratios for disease free survival in the adjuvant therapy after curative resection NMA-2. Relative effects in both direct and combined hazard ratios (HR) and 95% Credible Intervals (95% CrI) are shown to observe possible discrepancies between evidence derived from direct comparisons (top of each cell) and evidence derived from both direct and indirect comparisons (bottom of each cell). All *z*-tests to compare two treatments were performed two-sided. * *p* < 0.05. Abbreviations: A = anthracycline; ATr = anthracycline-based triplet; C = cisplatin; E = etoposide; F = fluoropyrimidine; I = irinotecan; M = mitomycin C; Obs = observation; Ox = oxaliplatin; OxFpr = doublet oxaliplatin with an one year treatment with a fluoropyrimidine; RCh = chemoradiotherapy; T = taxane.

Supplementary Tables: please find Table S1–S3 at additional excel file.

References

1. Tsuburaya, A.; Yoshida, K.; Kobayashi, M.; Yoshino, S.; Takahashi, M.; Takiguchi, N.; Tanabe, K.; Takahashi, N.; Imamura, H.; Tatsumoto, N.; et al. Sequential paclitaxel followed by tegafur and uracil (UFT) or S-1 versus UFT or S-1 monotherapy as adjuvant chemotherapy for T4a/b gastric cancer (SAMIT): A phase 3 factorial randomised controlled trial. *Lancet Oncol.* **2014**, *15*, 886–893, doi:10.1016/S1470-2045(14)70025-7.

2. Ter Veer, E.; Ngai, L.L.; Valkenhoef, G.V.; Mohammad, N.H.; Anderegg, M.C.J.; van Oijen, M.G.H.; van Laarhoven, H.W.M. Capecitabine, 5-fluorouracil and S-1 based regimens for previously untreated advanced oesophagogastric cancer: A network meta-analysis. *Sci. Rep.* **2017**, *7*, 7142, doi:10.1038/s41598-017-07750-3.

3. Nitti, D.; Wils, J.; Dos Santos, J.G.; Fountzilas, G.; Conte, P.F.; Sava, C.; Tres, A.; Coombes, R.C.; Crivellari, D.; Marchet, A.; et al. Randomized phase III trials of adjuvant FAMTX or FEMTX compared with surgery alone in resected gastric cancer. A combined analysis of the EORTC GI Group and the ICCG. *Ann. Oncol.* **2006**, *17*, 262–269.

4. Tsujinaka, T.; Shiozaki, H.; Inoue, M.; Furukawa, H.; Hiratsuka, M.; Kikkawa, N.; Takami, M.; Suzuki, T.; Monden, M. Evaluation of effectiveness of chemotherapy in patients with gastric cancer after curative resection. *Int. J. Clin. Oncol.* **2000**, *5*, 372–379.

5. Neri, B.; Cini, G.; Andreoli, F.; Boffi, B.; Francesconi, D.; Mazzanti, R.; Medi, F.; Mercatelli, A.; Romano, S.; Siliani, L.; et al. Randomized trial of adjuvant chemotherapy versus control after curative resection for gastric cancer: 5-year follow-up. *Br. J. Cancer* **2001**, *84*, 878–880.

6. Krook, J.E.; O’Connell, M.J.; Wieand, H.S.; Beart, R.W., Jr.; Leigh, J.E.; Kugler, J.W.; Foley, J.F.; Pfeifle, D.M.; Twito, D.I. A prospective, randomized evaluation of intensive-course 5-fluorouracil plus doxorubicin as surgical adjuvant chemotherapy for resected gastric cancer. *Cancer* **1991**, *67*, 2454–2458.

7. Park, S.H.; Lee, W.K.; Chung, M.; Lee, Y.; Han, S.H.; Bang, S.M.; Cho, E.K.; Shin, D.B.; Lee, J.H. Paclitaxel versus docetaxel for advanced gastric cancer: A randomized phase II trial in combination with infusional 5-fluorouracil. *Anticancer Drugs* **2006**, *17*, 225–229.

8. Ter Veer, E.; Haj Mohammad, N.; van Valkenhoef, G.; Ngai, L.L.; Mali, R.M.A.; Anderegg, M.C.; van Oijen, M.G.H.; van Laarhoven, H.W.M. The Efficacy and Safety of First-line Chemotherapy in Advanced Esophagogastric Cancer: A Network Meta-analysis. *J. Natl. Cancer. Inst.* **2016**, *108*, doi:10.1093/jnci/djw166.

9. Fuchs, C.S.; Tepper, J.E.; Niedzwiecki, D.; Hollis, D.; Mamon, H.J.; Swanson, R.; Haller, D.G.; Dragovich, T.; Alberts, S.R.; Bjarnason, G.A.; et al. Postoperative adjuvant chemoradiation for gastric or gastroesophageal junction (GEJ) adenocarcinoma using epirubicin, cisplatin, and infusional (CI) 5-FU (ECF) before and after CI 5-FU and radiotherapy (CRT) compared with bolus 5-FU/LV before and after CRT: Intergroup trial CALGB 80101. *J. Clin. Oncol.* **2011**, *29*, 4003–4003, doi:doi:10.1200/jco.2011.29.15_suppl.4003.


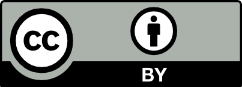
© 2019 by the authors. Licensee MDPI, Basel, Switzerland. This article is an open access article distributed under the terms and conditions of the Creative Commons Attribution (CC BY) license (http://creativecommons.org/licenses/by/4.0/).
